# Prompt engineering a large language model with evidence-based persuasive features to improve confidence in mental health professionals: a pilot randomized experiment

**DOI:** 10.3389/fpubh.2026.1909370

**Published:** 2026-07-27

**Authors:** Ang Li, Shi-Ting Yao, Bi-Xian Chen, Xin-Yu Li, Sai-Ya Guo

**Affiliations:** 1 Department of Psychology, Beijing Forestry University, Beijing, China; 2 School of Foreign Languages, Beijing Forestry University, Beijing, China

**Keywords:** confidence in mental health professionals, depression help-seeking attitude, large language model, prompt engineering, psychoeducation

## Abstract

**Background:**

Depression carries a heavy global burden, yet treatment gaps persist largely because individuals lack confidence in mental health professionals. Psychoeducation can shift these beliefs, but scaling persuasive messages is difficult. Large language models (LLMs) offer a scalable solution, though the specific text-based features that make LLM-generated psychoeducation persuasive remain unidentified. This study identified these features and tested their integration into an LLM prompt to shift confidence in mental health professionals.

**Methods:**

In Phase 1, 168 participants rated text pairs contrasting high versus low levels of four candidate features. In Phase 2, 40 participants were randomized to read psychoeducational passages generated by either a prompt incorporating the retained features (*n* = 20) or a baseline prompt (*n* = 20). The primary outcome was pre-to-post change in confidence in mental health professionals.

**Results:**

Source credibility, argument quality, and processing fluency significantly boosted both perceived credibility and persuasiveness (all *p* < 0.01); affective warmth and empathy was excluded. Participants reading feature-engineered texts showed greater improvement in attitudes than the control group (*t*(38) = 3.37, *p* = 0.002, *d* = 1.07). Controlling for baseline scores, the group effect remained significant (*F*(1,37) = 9.31, *p* = 0.004, partial *η*^2^ = 0.20).

**Conclusion:**

Strategically prompt-engineered LLM outputs incorporating empirically selected persuasive features significantly improve confidence in mental health professionals. This pilot study provides a preliminary evidence-based framework that may inform scalable, LLM-powered public mental health interventions.

## 1 Introduction

Depressive disorders affect approximately 332 million people globally and represent a leading cause of years lived with disability (1). All major types of structured psychotherapies are efficacious in treating depression (2), yet fewer than half of those experiencing depression seek professional care. In low- and middle-income countries, the treatment gap can exceed 80% (3). Even in well-resourced health systems, systemic and psychological barriers delay or prevent help-seeking (4–7).

Among these barriers, help-seeking attitudes offer a tractable target for intervention. The construct is multidimensional, comprising recognition of the need for psychological help, stigma tolerance, interpersonal openness, and confidence in mental health professionals (8, 9). Within this broader structure, confidence in mental health professionals stands out as a core component. Fischer and Turner (8) originally identified this confidence as a distinct attitudinal factor that directly underlies help-seeking behavior. More recently, Aldhamri (10) found that a lack of confidence in mental health professionals was the most prominent barrier to seeking psychological help among university students and significantly predicted intentions to use telemental health services. Similarly, Mackenzie et al. (11) demonstrated that help-seeking propensity (a construct reflecting belief in the utility of professional help) was the strongest attitudinal predictor of intentions to consult a professional. A meta-analysis by Kumpasoğlu et al. (12) documented a robust association between perceived therapist credibility and treatment outcomes (*r* = 0.35). Without a basic belief in a therapist’s competence and trustworthiness, other positive shifts in help-seeking attitudes may fail to translate into actual service use. Although the centrality of confidence in mental health professionals is well established, its measurement has been a subject of ongoing methodological discussion. Fischer and Turner’s (8) original 9-item Confidence subscale and the widely used shortened version (13) have demonstrated robust psychometric properties across diverse populations. However, in intervention research with a brief outcome assessment window, researchers often face a trade-off between scale length and participant burden, particularly when attitudes are captured immediately after stimulus exposure. In experimental research where attitude measurement occurs immediately after brief stimulus exposure, the use of single-item global measures is a pragmatic choice that has been discussed in the methodological literature (14). However, such measures are generally acknowledged to capture only the global evaluative component of the construct, leaving aside its more nuanced facets such as perceived competence, trustworthiness, and openness to professional influence. This limitation is especially relevant when working with LLM-generated materials, which may differentially affect these facets. The present study therefore adopts a single-item confidence measure as a pragmatic first step in an experimental framework, while acknowledging that future work will need to incorporate validated multi-item scales to disaggregate the specific dimensions of confidence that are most amenable to LLM-based persuasion. Confidence in mental health professionals therefore represents a key leverage point in public health interventions designed to promote professional help-seeking.

Psychoeducation can improve help-seeking attitudes when messages are accurate, engaging, and framed effectively (15, 16). Traditional delivery methods, such as printed brochures or static websites, face distinct limitations. Updating content is slow, personalization is minimal, and production costs accumulate. These constraints have prompted interest in computational approaches to generating health communication materials. This is not to suggest that traditional methods are obsolete; printed materials and static websites remain valuable for populations with limited digital literacy or internet access, and they avoid the risks of algorithmic bias and hallucination associated with LLM-generated content. Nonetheless, the scalability and adaptability of LLMs offer a complementary pathway that, if carefully harnessed, could extend the reach of evidence-based psychoeducation.

Large language models (LLMs) provide a new avenue for mental health communication. Models like GPT and DeepSeek can produce fluent, logically coherent texts on mental health topics in seconds. Prompt engineering—the design of systematic instructions that constrain an LLM’s output characteristics—enables the reliable incorporation of evidence-based communication strategies. Rather than leaving persuasive quality to chance, researchers can directly specify the linguistic and argumentative features they require.

The rapid spread of LLM applications in mental health is well documented (17, 18). Prompt engineering has become a core method for tailoring general-purpose LLMs to sensitive contexts, drawing on techniques such as few-shot prompting and instruction tuning (19). Advanced work has proposed layered prompt architectures that embed evidence-based therapeutic models directly into dialogue generation (20). At the same time, the application of LLMs to mental health communication is accompanied by significant concerns that must temper enthusiasm. First, LLMs are prone to generating factually incorrect or clinically misleading content, a phenomenon often referred to as hallucination, which in the context of depression psychoeducation could inadvertently reinforce maladaptive beliefs or provide unsafe advice (21). Second, LLMs can reproduce and amplify biases present in their training data, including racial, gender, and cultural stereotypes, and such biases can undermine the credibility of mental health messages among marginalized groups (22). Third, the simulated empathy and warmth that LLMs can generate may create a “therapeutic illusion” that masks the model’s lack of genuine understanding, raising ethical questions about the use of automated persuasion in vulnerable populations (23). These challenges underscore the necessity of moving beyond general-purpose prompt engineering to protocols that are explicitly grounded in evidence-based persuasive strategies and clinically traceable frameworks. They also highlight the importance of systematic evaluation—not only of whether LLM-generated messages are fluent and engaging, but of whether they are accurate, non-stigmatizing, and capable of producing genuine rather than illusory attitude change.

Despite these technical advances, empirical studies that identify which textual features make LLM-generated psychoeducation genuinely persuasive remain sparse. Most published evaluations compare LLM outputs with human-written materials on dimensions such as quality and empathy (24), without isolating specific persuasive mechanisms. The literature consistently identifies the absence of standardized, clinically grounded evaluation frameworks as a major gap. Hua et al. (18) note that most studies rely on *ad hoc* human assessments that do not capture therapeutic or persuasive quality. Cho et al. (17) reported that among 20 reviewed mental health chatbots, none had undergone randomized controlled trials measuring theory-driven behavior change. Priyadarshana et al. (19) observe that prompt engineering for mental health relies heavily on clinical intuition rather than systematic experimental evidence. Similarly, Chen et al. (25) argued that clinically traceable LLM outputs are essential for AI-assisted mental health applications, highlighting the importance of grounding generated content in verifiable diagnostic and therapeutic frameworks rather than relying solely on surface-level fluency.

Current prompt engineering practices for public health messaging are guided largely by general writing heuristics. Health communication research suggests that a message’s impact is contingent upon its specific features (26, 27). Dual-process theories of persuasion, particularly the Elaboration Likelihood Model (28), predict that both central-route features (e.g., argument quality) and peripheral-route features (e.g., source credibility, affective warmth and empathy, processing fluency) can shift attitudes. However, the same features can also backfire when they are perceived as manipulative or incongruent with the message recipient’s expectations. For instance, excessive affective warmth in a health message may activate suspicion about the communicator’s motives, and highly fluent processing may lead to shallow encoding that dissipates rapidly. Therefore, the integration of persuasive features into LLM prompts must be done with careful attention to the specific combination and intensity of features, and the persuasive effects must be empirically verified rather than assumed. The critical gap in the LLM literature is the shortage of controlled experiments that manipulate such textual features within prompt-engineered outputs and measure their persuasive effects on mental health attitudes. Moreover, the few existing experimental studies have predominantly assessed immediate self-reported attitudes, leaving the durability of persuasion effects and their translation into actual help-seeking behavior largely unexplored in the current evidence base. This restricts the conclusions that can be drawn from current evidence. The present study, like the works it builds upon, is limited to assessing immediate attitude change and does not address durability or behavior. However, the field requires a stepwise approach in which feature identification and immediate attitude change must first be established before longitudinal designs and behavioral endpoints become interpretable.

Addressing this gap, the present study pursued three aims: (a) to screen four theoretically grounded persuasive features (source credibility, argument quality, affective warmth and empathy, and processing fluency) using human ratings of credibility and persuasiveness; (b) to integrate retained features into a prompt engineering protocol applied to DeepSeek-V3.2, generating a set of depression psychoeducational texts; and (c) to evaluate, through a randomized between-subjects experiment, whether texts produced by this feature-integrated prompt yield greater improvements in confidence in mental health professionals than texts from a baseline prompt.

## 2 Methods

### 2.1 Ethics

The study protocol was approved by the Institutional Review Board of the Institute of Psychology, Chinese Academy of Sciences (Protocol No. H23141) and was conducted in accordance with the Declaration of Helsinki. All participants provided electronic informed consent.

### 2.2 Phase 1: Persuasive feature screening

#### 2.2.1 Participants

A sample of 168 general-population adults was recruited through online platforms (age: 23.63 ± 5.58 years; men: 56, women: 112). Inclusion criteria were age 18 years or older and proficient Chinese reading ability.

#### 2.2.2 Design and materials

Based on the Elaboration Likelihood Model of Persuasion, four persuasive features were evaluated. Each feature was operationalized as a matched pair of psychoeducational texts addressing depression, including a pro-version designed to be high on the feature and a con-version low on it.

Source credibility was manipulated as the presence versus absence of an authoritative source cue. In the high-credibility condition, the message opened with an explicit attribution to a highly expert and trustworthy source (e.g., World Health Organization). In the low-credibility condition, the message omitted any reference to a source. Apart from this opening statement, the two versions were lexically identical.

Argument quality was operationalized as the presence versus absence of substantive reasoning. In the strong-argument condition, the message offered a logically structured rationale in support of the central claim, including causal links and specific factual grounds. In the weak-argument condition, the identical claim was presented as a bare assertion, with no supporting reasoning or evidence.

Affective warmth and empathy were manipulated via the emotional tone of language and the degree of interpersonal validation. In the high-warmth condition, the message used emotionally expressive vocabulary, employed first- and second-person pronouns, and included explicit statements validating the reader’s emotional difficulties. In the low-warmth condition, the message was written in a neutral, impersonal style, using only third-person constructions and factual descriptors. Sentence length and syntactic complexity were closely matched between the two conditions to avoid confounding with processing fluency.

Processing fluency was manipulated through the phonological and rhythmic fluency of the text, holding propositional content constant. In the high-fluency condition, sentences were constructed with a conspicuous rhythmic cadence and a clear end-rhyme scheme. These sentences relied exclusively on high-frequency, familiar vocabulary and avoided figurative language. In the low-fluency condition, the equivalent content was expressed through sentences lacking rhythmic regularity, employing longer, syntactically complex structures and lower-frequency words.

Each text covered identical depression psychoeducational content. A clinical psychologist reviewed all materials for accuracy. To minimize the risk that observed differences could be attributed to idiosyncratic text characteristics rather than the intended feature manipulation, the pro- and con-versions within each feature pair were matched on propositional content, overall text length, and, where feasible, syntactic complexity. The clinical psychologist also verified that the two versions within each pair were equivalent in factual coverage and did not differ on dimensions unrelated to the target feature (e.g., the strong- and weak-argument versions conveyed identical claims and differed only in the presence vs. absence of supporting reasoning).

#### 2.2.3 Measures

After reading each text, participants rated two items on five-point Likert scales (1 = strongly disagree, 5 = strongly agree): “I find this text very credible” and “This text could persuade me to improve my attitude toward depression.” These items capture the trustworthiness of the message and its perceived capacity to shift personal attitudes.

#### 2.2.4 Procedure

Participants completed the study online. After providing informed consent, they viewed the eight texts in randomized order. Each text was displayed on a separate screen followed by the two rating items. No time limit was imposed. A feature was retained for Phase 2 only if the pro-version scored significantly higher than the con-version on both credibility and persuasiveness at *p* < 0.05 by paired-samples *t*-tests.

#### 2.2.5 Data analysis

Paired-samples *t*-tests (two-tailed, *α* = 0.05) and the corresponding Cohen’s *d* effect sizes were computed for each feature using IBM SPSS Statistics 27.

#### 2.2.6 Transparency and reproducibility

All eight text pairs used in Phase 1 (pro- and con- versions for each of the four persuasive features) are provided in full in Appendix A. The texts were originally written in Chinese by the research team, based on the operational definitions described in Section 2.2.2, and were reviewed by a clinical psychologist to ensure factual accuracy and alignment with current clinical guidelines for depression psychoeducation. The English versions presented in Appendix A are the authors’ translations, produced through forward translation by a bilingual member of the research team and independently checked for accuracy by a second bilingual author. Any discrepancies between the original Chinese texts and the back-translations were resolved through discussion.

### 2.3 Phase 2: Prompt engineering and randomized intervention

#### 2.3.1 Prompt engineering and LLM configuration

DeepSeek-V3.2 was accessed via its application programming interface on December 2025. Generation parameters were set to temperature = 0.7 and top-*p* = 0.9 and held constant across both the experimental and baseline conditions. The three features retained from Phase 1—source credibility, argument quality, and processing fluency—were incorporated simultaneously into a single system prompt through iterative refinement. The development process began with a draft prompt specifying: “Act as an experienced expert in the popularization of psychology.” The research team added requirements for a logically structured rationale and fluent, rhythmic prose. Outputs were manually inspected; when the model failed to demonstrate sources reliability, produce high-quality arguments, or maintain rhythmic regularity, additional constraints were added.

The final system prompt (see Appendix B) instructed the model to (a) assume the role of an experienced psychology popularization expert and anchor all statements in authoritative sources and high-quality research, (b) systematically correct common public misconceptions by scientifically explaining why such beliefs are erroneous, and (c) employ jargon-free, fluent language and close with a semantically concise, rhythmically patterned rhyming summary. Based on this prompt, five depression psychoeducational topics were generated: causes and risk factors, diagnosis and assessment, treatment and intervention, prevention and self-care, and social support and recovery. Each passage was reviewed for psychological accuracy.

For the baseline prompt, a minimal instruction was used: “Write a psychoeducational text about depression on topic X” with the same five topics. Outputs were approximately equal in length.

#### 2.3.2 Participants

Forty new participants were recruited through online platforms (age: 24.28 ± 7.30; men: 18, women: 22), with no overlap with the Phase 1 sample. Inclusion criteria remained age ≥ 18 years and Chinese reading proficiency. Participants were randomly assigned to the Prompt-Engineered Group (PEG, *n* = 20) or Baseline Prompt Group (BPG, *n* = 20).

#### 2.3.3 Measures

The primary outcome was a single-item measuring confidence in mental health professionals, assessed pre- and post-intervention: “If I were experiencing depression now, I believe a psychological expert could help me treat it.” Responses were provided on a four-point Likert scale (1 = strongly disagree, 4 = strongly agree).

We opted for a single-item measure in this initial pilot investigation for several reasons. First, the item targets a highly specific and concrete belief—confidence in the ability of a psychological expert to provide effective treatment—rather than a broad, multidimensional latent construct. Methodological research has demonstrated that single-item measures can perform comparably to multi-item scales when the construct being measured is sufficiently narrow and unambiguous to the respondent (14, 29). Second, the construct of confidence in mental health professionals was originally identified as a distinct, unidimensional factor within the Attitudes Toward Seeking Professional Psychological Help scale (8), and our item was designed to capture that specific factor without the additional semantic loading that accompanies items measuring stigma tolerance or interpersonal openness. Third, in a randomized experimental design where the primary interest is in between-group differences in change scores, the key psychometric requirement is sensitivity to treatment-induced change rather than broad content coverage. A focused single item reduces common method variance that can arise when lengthy scales are administered immediately before and after a brief reading intervention.

#### 2.3.4 Procedure

After consent and baseline assessment, participants read all five texts corresponding to their assigned condition, presented sequentially on screen. Reading was self-paced. Immediately following the final text, the post-intervention measure was administered. No other activities occurred between pre- and post-assessment.

#### 2.3.5 Data analysis

An independent-samples *t*-test compared change scores between groups. A one-way ANCOVA was conducted with post-test as the dependent variable, group as the fixed factor, and pre-test as the covariate. Homogeneity of regression slopes was tested via the Group × Pre-test interaction; Levene’s test assessed equality of error variances. Within-group pre-post comparisons used paired-samples *t*-tests. Effect sizes reported are Cohen’s *d* (for *t*-tests) and partial *η*^2^ (for ANCOVA). Alpha was set at 0.05, and the ANCOVA group comparison used Bonferroni correction. Analyses were performed in IBM SPSS Statistics 27.

#### 2.3.6 Transparency and reproducibility

To facilitate replication and independent evaluation, we report the following details. The LLM used was DeepSeek-V3.2, accessed via its application programming interface (API) on December 2025. Generation parameters were held constant across conditions: temperature = 0.7, top-*p* = 0.9. The exact system prompt for the experimental condition and the minimal prompt for the baseline condition are provided in Appendix B. All ten LLM-generated passages (five from the experimental prompt and five from the baseline prompt, corresponding to the five depression psychoeducational topics) are provided in full in Appendix C. The texts were originally generated in Chinese. The English versions presented in Appendix C are the authors’ translations, produced through forward translation by a bilingual member of the research team and independently checked for accuracy by a second bilingual author. To verify the factual accuracy of the LLM-generated content, a clinical psychologist reviewed all passages against current clinical guidelines (including ICD-11 and DSM-5 diagnostic criteria and evidence-based treatment recommendations). When factual inaccuracies or clinically misleading statements were identified, the prompt was iteratively refined and the text regenerated until the output met the accuracy criterion. The final versions included in the Supplementary material are those that passed this clinical review.

## 3 Results

### 3.1 Phase 1: Persuasive feature screening

Three features (source credibility, argument quality, and processing fluency) met the retention criterion of statistically significant differences in both credibility and persuasiveness ratings, with pro-versions consistently rated higher (all *p* < 0.01) (see Table 1). Affective warmth and empathy yielded a significant persuasiveness advantage for the pro-version, but the credibility difference was not significant. This feature was therefore excluded from Phase 2 prompt engineering.

**Table 1:** Ratings for pro- and conversions of each persuasive feature (*n* = 168).

Features	Evaluation	Pro *M* (SD)	Con *M* (SD)	*t*(167)	*p*	*d*
Source credibility	Credibility	4.18 (0.75)	3.77 (1.02)	−4.83	<0.001	−0.37
	Persuasiveness	4.03 (0.70)	3.76 (0.93)	−3.63	<0.001	−0.28
Argument quality	Credibility	4.11 (0.89)	3.90 (0.91)	−3.11	0.002	−0.24
	Persuasiveness	4.06 (0.84)	3.83 (0.86)	−3.52	<0.001	−0.27
Affective warmth & empathy	Credibility	4.11 (0.83)	4.05 (0.73)	−0.93	0.355	−0.07
	Persuasiveness	4.06 (0.83)	3.92 (0.58)	−2.30	0.023	−0.18
Processing fluency	Credibility	4.00 (0.73)	3.80 (0.94)	−2.88	0.004	−0.22
	Persuasiveness	4.00 (0.83)	3.76 (0.90)	−3.66	<0.001	−0.28

Because four features were each tested on two dependent measures (credibility and persuasiveness), eight paired-samples *t*-tests were conducted in Phase 1. Although these tests were planned *a priori* based on the Elaboration Likelihood Model and each addressed a distinct theoretical question, we evaluated the robustness of our screening decisions against a conservative Bonferroni-adjusted alpha of 0.00625 (0.05/8). All three retained features remained significant at this corrected level for both credibility and persuasiveness (all *p* ≤ 0.004). Affective warmth and empathy remained non-significant for credibility (*p* = 0.355) and would not survive correction for persuasiveness (*p* = 0.023). Thus, the pattern of retention and exclusion is unchanged under the corrected threshold.

### 3.2 Phase 2: Randomized intervention

An independent-samples *t*-test confirmed that the random assignment produced comparable groups at baseline, with no significant difference in pre-intervention attitude scores between the PEG and the BPG (*t*(38) = −1.40, *p* = 0.170, *d* = −0.44). Table 2 presents the pre-test and post-test means and standard deviations for both groups. No follow-up assessment was conducted, so the durability of the observed change remains unknown.

**Table 2:** Pre-test and post-test scores on confidence in mental health professionals by group.

Group	*n*	Pre-test *M* (*SD*)	Post-test *M* (*SD*)	Change *M* (*SD*)	*t*(19)	*p*
PEG	20	3.15 (0.75)	3.60 (0.60)	0.45 (0.69)	−2.93	0.009
BPG	20	3.45 (0.61)	3.25 (0.55)	−0.20 (0.52)	1.71	0.104

PEG, Prompt-Engineered Group; BPG, Baseline Prompt Group. Scale range: 1–4. Within-group *p*-values are from paired-samples *t*-tests (two-tailed).

Change score analysis revealed a significant group difference. PEG participants showed a mean increase of 0.45 (SD = 0.69) and BPG participants a mean decrease of −0.20 (SD = 0.52), *t*(38) = 3.37, *p* = 0.002, *d* = 1.07, 95% CI [0.40, 1.72].

Assumptions for ANCOVA were satisfied. The Group × Pre-test interaction was non-significant, *F*(1,36) = 0.34, *p* = 0.563, partial *η*^2^ = 0.009, confirming homogeneity of regression slopes. Levene’s test indicated equal error variances, *F*(1,38) = 0.87, *p* = 0.358. The ANCOVA demonstrated a significant main effect of group, *F*(1,37) = 9.31, *p* = 0.004, partial *η*^2^ = 0.20. Adjusted post-test means (controlling for pre-test) were 3.67 (SE = 0.11) for PEG and 3.18 (SE = 0.11) for BPG, with a mean difference of 0.49, 95% CI [0.16, 0.81], *p* = 0.004. The covariate (pre-test) was also significant, *F*(1,37) = 14.95, *p* < 0.001, partial *η*^2^ = 0.29.

Within-group analyses confirmed that the overall group effect was attributable to change in the PEG condition. PEG participants showed a significant pre-post increase, *t*(19) = −2.93, *p* = 0.009, *d* = −0.66. The BPG change was non-significant, *t*(19) = 1.71, *p* = 0.104, *d* = 0.38.

## 4 Discussion

This study tested whether a systematic, evidence-based approach to LLM prompt engineering could strengthen depression psychoeducational texts and shift recipients’ confidence in mental health professionals. The two-phase design yielded findings that are encouraging for the development of digital psychoeducational materials, while remaining preliminary with respect to broader help-seeking outcomes.

First, the feature screening demonstrated that not all intuitively appealing persuasive strategies survive empirical scrutiny. Source credibility, argument quality, and processing fluency each independently enhanced both perceived trustworthiness and self-reported persuasive potential. Affective warmth and empathy, despite its persuasiveness advantage, did not concurrently elevate credibility ratings. This dissociation is instructive. Emotionally warm language may signal compassion and increase subjective persuasiveness through an affective pathway, but it may simultaneously fail to reinforce the perception of informational authority (30–33). The result echoes findings from health communication research showing that emotional appeals require careful calibration: when overlaid onto ostensibly informational content, they can read as formulaic rather than genuinely supportive (34, 35). The exclusion of this feature does not imply that warmth is irrelevant to mental health communication broadly. It suggests that when the goal is to build credibility-weighted persuasion in brief psychoeducational texts, fluency and argumentation prove more reliable.

The three retained features map cleanly onto dual-process models of persuasion. Source credibility operates through the peripheral route, functioning as a heuristic cue that reduces the cognitive effort required to evaluate message validity (28). Explicitly invoking professional authority in the prompt allowed the LLM to embed identity markers that signaled expertise without overstatement. Argument quality engages the central route. The engineered prompt instructed the LLM to systematically correct common public misconceptions by scientifically explaining why such beliefs are erroneous and to present a coherent chain of reasoning. This operationalization ensures that each text offers readers substantive evidence and logical coherence to scrutinize. Prior research confirms that arguments possessing such characteristics are more persuasive than those that merely assert a claim without supporting evidence (36). Processing fluency occupies an intermediate position. Fluent text feels easier to process, and this metacognitive ease is misattributed to properties of the message itself—a phenomenon termed the truth-by-fluency effect (37). Rhythmic, clear prose is a genuine persuasive lever.

Second, the randomized experiment confirmed that integrating these features through systematic prompt engineering produced measurable attitudinal change. It is worth noting that the Phase 1 screening already established that each of the three retained features independently enhanced both perceived credibility and persuasiveness in isolation. The Phase 2 outcome is therefore consistent with the interpretation that these features, when combined in a single prompt, collectively drove the observed group difference, even though the present design cannot decompose their individual contributions. Participants who read the PEG texts showed a significant pre-to-post increase in the single-item measure of confidence in mental health professionals, with a within-group effect of moderate size (Cohen’s *d* = −0.66) and an ANCOVA-derived between-group effect in the moderate-to-large range (partial *η*^2^ = 0.20). The baseline prompt texts produced no pre-post change. This null finding illustrates a point often obscured in discussions of LLM capabilities: generation fluency and superficial coherence do not automatically confer persuasive impact. The LLM produced competent baseline texts, but competence was insufficient to alter a pre-existing belief about professional help. Persuasive architecture had to be engineered into the prompt explicitly.

The results extend prior LLM-and-mental-health research by experimentally isolating text features that generate persuasion, rather than evaluating LLM outputs as holistic entities. This approach moves the field toward a component-based understanding of LLM-generated health communication. For depression psychoeducation specifically, knowing that source credibility, argument quality, and processing fluency constitute necessary ingredients provides a falsifiable blueprint for future prompt designs. Nevertheless, given the small sample and the immediate, single-item nature of the outcome, these findings should be regarded as hypothesis-generating and interpreted with appropriate caution.

The public health implications of these findings are substantial. Mental health organizations and public health agencies increasingly incorporate LLMs into content production pipelines to address population-level treatment gaps. An evidence-based prompt engineering protocol reduces reliance on individual clinical intuition. It enables the scalable generation of psychoeducational content that maintains consistency across topics, languages, and target populations, provided the core persuasive features are retained. If the observed short-term shift in confidence in mental health professionals can be replicated in larger samples and shown to influence downstream behavior, feature-engineered LLM outputs could eventually serve as a low-cost, highly accessible component of population-level dissemination strategies. The five-topic structure used in this study demonstrates that feature-engineered text can be generated across a comprehensive psychoeducational sequence without loss of persuasive integrity.

Several limitations must be weighed. First, the Phase 2 sample size (*n* = 40) is small, although the effect sizes were large enough to achieve statistical significance and provide informative estimates for power calculations in subsequent trials. Second, a further limitation concerns the nature of the control condition. The baseline prompt was a minimal, generic instruction, whereas the experimental prompt combined multiple requirements, including the assumption of an expert role, source grounding, misconception correction, structured argumentation, and fluency constraints. The observed group difference therefore reflects the aggregate effect of the integrated feature set rather than the unique contribution of any single persuasive element. Although Phase 1 demonstrated that source credibility, argument quality, and processing fluency each independently enhance perceived credibility and persuasiveness when tested in isolation, the present Phase 2 design cannot isolate their causal roles when they are combined in a single prompt. It is possible that the mere increase in prompt specificity and detail, rather than the particular persuasive features it encodes, contributed to the observed effect. Future research should employ factorial designs that systematically vary the presence or absence of each feature while holding other prompt characteristics constant across conditions. Such designs would allow the field to determine whether the persuasive effects observed here are attributable to the specific features identified in Phase 1 or to more general properties of elaborated prompts. Third, the primary outcome was assessed immediately after reading using a single item. This single-item measure cannot capture the multi-dimensional nature of help-seeking attitudes fully, and the absence of any follow-up means that durability of the attitude change is entirely unknown. It is possible that the immediate post-test improvement observed in the PEG condition reflects a transient priming or recency effect rather than a lasting shift in belief; without follow-up data, we cannot distinguish between these possibilities. Because the present study measured only immediate self-reported confidence, we make no claim that these short-term attitude changes translate into reduced treatment gaps; this remains an empirical question for future research with behavioral endpoints and longitudinal designs. In this respect, our study joins other recent proof-of-concept investigations in the LLM-and-mental-health domain that have similarly relied on small samples and immediate outcomes. For instance, Chen et al. (38), in their exploratory work on wearable-sensing and retrieval-augmented LLMs for personalized mental health dialogue, appropriately characterized their findings as preliminary and called for larger-scale validation before any claims of clinical utility can be made. We echo that caution here. The reliance on a single item to measure confidence in mental health professionals further precludes assessment of other important dimensions such as stigma tolerance, interpersonal openness, and actual behavioral intentions. Future research should employ validated multi-item instruments to capture the full attitudinal construct with greater reliability and breadth. Additionally, we recommend that subsequent studies incorporate at least one delayed follow-up assessment (e.g., at one week and one month post-intervention) to evaluate whether the attitudinal shifts induced by feature-engineered psychoeducational texts persist beyond the immediate reading context. Importantly, the present study provides no evidence of clinical impact, actual behavior change, or public health effectiveness. It is a laboratory-based pilot investigation of immediate attitudinal response to LLM-generated psychoeducational text in a general-population convenience sample. The translation of the observed short-term shift in confidence in mental health professionals into meaningful clinical outcomes—such as increased help-seeking behavior, service utilization, or treatment engagement—remains an entirely open empirical question that requires future research with behavioral endpoints and clinical populations. Fourth, participants were drawn from a general population convenience sample, and results cannot be directly generalized to individuals with current clinical depression, for whom baseline help-seeking beliefs may be more entrenched. Fifth, demand characteristics are a concern whenever participants read persuasive material and self-report attitudes; the non-significant change in the BPG condition partially mitigates this concern, as both groups underwent identical procedures. Sixth, only one LLM (DeepSeek-V3.2) was tested with one set of generation parameters, limiting generalizability to other models. Finally, affective warmth and empathy was excluded based on a stringent dual-significance criterion. In longer-form interventions or repeated-exposure designs with clinical populations, affective warmth and empathy might contribute to engagement in ways this screening protocol missed. The exclusion also raises a theoretical question about whether persuasive features interact when combined in a single text. It is possible that processing fluency and source credibility jointly amplify each other, or that affective warmth and empathy, when added as a fourth feature, could potentiate the effects observed here. These interactions warrant investigation in factorial experiments.

## 5 Conclusion

This pilot study provides initial evidence that incorporating empirically screened persuasive features into prompts may offer one systematic way to generate psychoeducational content. In this small sample, the features of source credibility, argument quality, and processing fluency were associated with a shift in immediate self-reported confidence, a shift not observed with the standard prompt. Given the sample size (*N* = 40), the single-item immediate outcome, and the absence of follow-up, these findings should be regarded as preliminary and hypothesis-generating. They do not establish a replicable method or durable attitude change. The integration of experimental persuasion research with prompt engineering suggests a direction for future work, but the present data only point to a possible template whose utility and generalizability remain to be tested.

## Data Availability

The raw data supporting the conclusions of this article will be made available by the authors, without undue reservation.
