# Supplementary material for: Prompt engineering a large language model with evidence-based persuasive features to improve confidence in mental health professionals: a pilot randomized experiment
Source: Front Public Health. 2026 Jul 27;14:1909370. doi: 10.3389/fpubh.2026.1909370 (PMC13453785; doi:10.3389/fpubh.2026.1909370)
Supplement: Supplementary file 1 [file Supplementary_file_1.docx]

**Appendix A: Eight text pairs in phase 1**

**(1) Source credibility**

**Pro-version:** According to the NHC, the combination of standardized antidepressant treatment and adjunctive psychotherapy is associated with substantially higher remission rates. (original Chinese: 根据国家卫健委的报告，规范使用抗抑郁药配合心理辅导，康复率可显著提高。)

**Con-version:** According to an online article, the combination of standardized antidepressant treatment and adjunctive psychotherapy is associated with substantially higher remission rates. (original Chinese: 网上文章说，规范使用抗抑郁药配合心理辅导，康复率可显著提高。)

**(2) Argument quality**

**Pro-version:** Some people believe that psychological problems can simply be "endured until they pass." This notion is mistaken, and such an approach does not genuinely resolve the underlying issues. Without timely intervention, negative emotions and symptoms may continue to accumulate and intensify, potentially precipitating more severe mental disorders. Only by seeking timely help can individuals better safeguard their mental health and alleviate the suffering of patients with depression. (original Chinese: 有人认为心理问题“忍一忍就过去了”，这种想法是错误的，这种做法无法真正解决问题。若不及时干预，负面情绪和症状可能持续累积、加重，甚至引发更严重的心理障碍。只有及时求助才能更好地维护心理健康，缓解抑郁症病人的痛苦。)

**Con-version:** Some people believe that psychological problems can simply be "endured until they pass." This notion is mistaken, and such an approach does not genuinely resolve the underlying issues. Only by seeking timely help can individuals better safeguard their mental health and alleviate the suffering of patients with depression. (original Chinese: 有人认为心理问题“忍一忍就过去了”，这种想法是错误的，这种做法无法真正解决问题，只有及时求助才能更好地维护心理健康，缓解抑郁症病人的痛苦。)

**(3) Affective warmth and empathy**

**Pro-version:** Those who say "depression is just being melodramatic" simply do not understand the cruelty of this suffering! (original Chinese: 那些说“抑郁症就是矫情”的人，根本不懂这种痛苦的残酷！)

**Con-version:** Those who say "depression is just being melodramatic" may be operating under a misconception. (original Chinese: 那些说“抑郁症就是矫情”的人，可能存在一定的误解。)

**(4) Processing fluency**

**Pro-version:** When sleep is broken, mood is low, and mornings come too soon, two weeks of suffering is a sign to heed. (original Chinese: 失眠早醒情绪糟，两周未好要警惕。)

**Con-version:** Sleep disturbance and depressed mood persisting for more than two weeks warrant clinical attention. (original Chinese: 持续两周以上的睡眠障碍和情绪低落需要引起重视。)

**Appendix B: The final prompt**

**(1) The prompt-engineered group:**

You are an experienced psychology popularization expert. Produce a single, coherent psychoeducational passage on *[specific depression-related topic]*, addressed to the general public. The passage must seamlessly weave all of the following requirements into one continuous narrative, without dividing the text into separate sections, bullet points, or labeled parts.

1. Source credibility and accuracy: Ground every claim in authoritative, verifiable sources—such as the World Health Organization (WHO), the American Psychiatric Association (APA), the Chinese Psychological Society (CAP), clinical manuals including the ICD-11 and DSM-5, and high-quality peer-reviewed research. Do not fabricate any information.

2. Misconception correction with scientific explanation: Provide detailed corrections of common public misunderstandings about the topic. For each misconception, articulate scientifically why the belief is erroneous and present the accurate conceptualization or evidence-based alternative.

3. Processing fluency and memorability: Use clear, jargon-free language and maintain a rigorous yet gentle tone. Conclude the passage with a semantically concise, rhythmically patterned rhyming summary that enhances retention and shareability. Refrain from employing literary devices such as metaphor or personification.

**(2) The baseline prompt group:**

Write a psychoeducational text about depression on *[specific depression-related topic]*

**Appendix C: LLM-generated passages in phase 2**

**(1) The prompt-engineered group:**

**① Topic on causes and risk factors:**

About Depression: Understanding, Recognizing, and Responding with Science

Depression is a common mental health condition. It is neither a passing low mood nor a sign of personal weakness, but a medical mood disorder that requires proper attention. It persistently affects how a person feels, thinks, and functions physically, causing significant interference with daily life, work, and interpersonal relationships. According to the World Health Organization, hundreds of millions of people worldwide are affected, and depression is one of the leading causes of disability globally.

I. Core Symptoms: More Than Just Feeling Low

Based on clinical descriptions in the ICD-11 (World Health Organization) and DSM-5 (American Psychiatric Association), the core feature of depression is a change in mood and functioning that differs from the individual's previous state and lasts for at least two weeks. The core symptoms fall into two broad categories.

1. Core affective symptoms (at least one must be present)

Persistent low mood: Feeling sad, empty, hopeless, or tearful for most of the day, nearly every day. This mood is not always triggered by a specific event and does not easily lift.

Markedly diminished interest or pleasure: A noticeable loss of interest in almost all activities, including hobbies, socializing, or leisure pursuits that were previously enjoyed. Little or nothing brings a sense of pleasure.

2. Other common associated symptoms

Fatigue or loss of energy: An unusual degree of tiredness and lack of energy that is not relieved by rest. Even small tasks feel extraordinarily effortful.

Cognitive changes: Difficulty concentrating, memory problems, and indecisiveness.

Diminished self-worth: Excessive or inappropriate feelings of guilt and worthlessness.

Somatic symptoms:

Sleep disturbance: difficulty falling asleep, staying asleep, or early-morning awakening; or, conversely, sleeping too much.

Significant change in appetite or weight: marked decrease or increase in appetite, leading to a notable weight change within a month.

Psychomotor changes: observable restlessness (agitation) or slowing of speech and movement (retardation).

Negative thoughts: Recurrent thoughts of death or suicide, which may in some cases be accompanied by a specific plan or attempts.

Important note: A person does not need to experience every symptom listed here for depression to be present. The combination of symptoms, their severity, and their duration vary from one individual to another. When several of these symptoms (especially the core affective ones) persist and cause significant distress or functional impairment, it warrants clinical attention.

II. Causes and Risk Factors: The Interplay of Multiple Forces

Contemporary science understands depression not as a condition with a single cause but as a product of a complex interaction among biological, psychological, and social-environmental factors. One can think of it as a lock that requires several keys to turn at the same time before it opens.

1. Biological factors

Brain chemistry: Dysfunction in neurotransmitter systems involved in mood regulation, such as serotonin, norepinephrine, and dopamine, has been implicated.

Genetic liability: Having a family history of depression raises an individual's risk. This does not mean depression is inevitable; it signals a heightened vulnerability, not a destiny.

Brain structure and function: Alterations in the structure or functional connectivity of certain brain regions, including the prefrontal cortex and hippocampus, may be observed.

Hormones and physical illness: Endocrine disorders (e.g., thyroid dysfunction), chronic pain, serious chronic medical conditions (e.g., cardiovascular disease, cancer), and neurological illnesses can all precipitate depressive symptoms.

2. Psychological factors

Temperament and personality traits: Long-standing tendencies toward low self-esteem, perfectionism, excessive self-criticism, or a pessimistic cognitive style.

Early life experiences: Childhood maltreatment, neglect, loss, or other traumatic events can increase vulnerability in adulthood.

Coping style: Reliance on less adaptive coping strategies, such as suppression or avoidance, in the face of stress.

3. Social-environmental factors and stress

Major life events: These are common triggers and include bereavement, relationship breakdown, unemployment, financial crisis, exposure to violence, or serious illness.

Chronic stress: Persistent pressures such as unrelenting work demands, ongoing family conflict, or serving as a caregiver for a seriously ill relative.

Inadequate social support: A lack of close, confiding relationships and the experience of loneliness and social isolation.

A crucial understanding: Developing depression is not a person's fault and is not a sign of weak willpower. Risk factors merely raise the probability, while protective factors—such as strong social support, healthy coping skills, and an active lifestyle—build psychological resilience. Recognizing these factors helps us appreciate the complexity of depression and points toward effective, scientifically grounded pathways of intervention.

III. Seeking Professional Help Is the First Step Toward Recovery

Depression is a treatable and manageable illness. Much like chronic conditions such as hypertension or diabetes, the vast majority of those affected can achieve significant relief from symptoms and restore normal social functioning through evidence-based interventions—whether psychotherapy, medication, or a combination of the two.

If you or someone you care about is experiencing the difficulties described above, the crucial step is to seek a professional evaluation from a psychiatrist or clinical psychologist. Doing so is not a sign of weakness but an act of responsibility and courage toward one's own health.

References and Authoritative Sources:

World Health Organization. International Classification of Diseases, 11th Revision (ICD-11).

American Psychiatric Association. Diagnostic and Statistical Manual of Mental Disorders, 5th Edition, Text Revision (DSM-5-TR).

Chinese Guidelines for the Prevention and Treatment of Depressive Disorders.

National Institute of Mental Health (NIMH) public education resources.

Original Chinese:

关于抑郁症：了解、识别与科学应对

抑郁症是一种常见的心理健康状况，它不仅仅是一时的“心情不好”或“脆弱”，而是一种需要被认真对待的医学上的情绪障碍。它会持续影响人的情绪、思维和身体功能，显著干扰日常生活、工作和人际关系。根据世界卫生组织（WHO）的统计，全球有数亿人受其影响，它是导致失能的主要原因之一。

一、 核心症状：不仅是情绪低落

根据世界卫生组织（ICD-11）和美国精神医学学会（DSM-5）的临床描述，抑郁症的核心特征通常表现为持续至少两周的、与先前不同的情绪和功能变化。核心症状可归纳为以下两类：

1. 核心情绪症状（至少具备其一）

持续的情绪低落：几乎整天感到悲伤、空虚、绝望或“想哭”，这种情绪并非总由具体事件引起，且难以自行排解。

兴趣或愉悦感丧失：对几乎所有活动都失去兴趣，即使是曾经非常热爱的爱好、社交或娱乐活动，也无法从中感受到快乐。

2. 其他常见相关症状

精力减退与疲劳：异常地疲乏无力，即使休息后也难以恢复，做小事也感觉非常费力。

认知功能变化：注意力难以集中，记忆力下降，做决定变得犹豫不决。

自我价值感降低：产生过度或不恰当的内疚感、无价值感。

躯体症状：

睡眠障碍：难以入睡、易醒或早醒，或相反地过度睡眠。

食欲/体重显著变化：食欲明显减退或暴增，导致体重在一个月内显著减轻或增加。

精神运动性变化：可被观察到的烦躁不安（激越）或言语动作迟缓（迟滞）。

消极念头：反复出现关于死亡、自杀的念头，有时可能有具体的自杀计划或尝试。

重要提示：并非有上述所有症状才可能是抑郁症。症状组合、严重程度和持续时间因人而异。如果上述症状中（特别是包含核心情绪症状）有数项持续存在，并造成痛苦或功能损害，就应引起重视。

二、 成因与风险因素：多重因素的共同作用

现代科学认为，抑郁症并非由单一原因造成，而是生物、心理与社会环境多重因素复杂交互的结果。这好比一把“锁”，可能需要多把“钥匙”同时转动才会打开。

1. 生物学因素

脑内化学物质失衡：研究发现，与情绪调节相关的神经递质（如血清素、去甲肾上腺素和多巴胺）功能紊乱可能有关。

遗传倾向：家族中有抑郁症病史，个体患病风险会增高。但这并非“注定”，它意味着易感性更高，而非必然性。

大脑结构与功能：某些脑区（如与情绪调节相关的额叶、海马体等）的结构或功能连接可能出现变化。

激素与躯体疾病：内分泌疾病（如甲状腺问题）、慢性疼痛、严重慢性病（如心脏病、癌症）或神经系统疾病也可能诱发抑郁症状。

2. 心理学因素

气质与性格特质：如长期倾向自卑、完美主义、过度自我批评或悲观思维模式。

早期经历：童年时期经历的虐待、忽视、丧失或其他创伤性事件，可能增加成年后的患病风险。

应对方式：面对压力时，习惯采用压抑、逃避等不成熟的应对策略。

3. 社会环境与应激因素

重大生活事件：这是常见的诱因，如亲人的丧失、婚姻或亲密关系破裂、失业、经济危机、遭受暴力或严重疾病。

长期慢性压力：如工作压力过载、长期家庭冲突、照顾重病家人等。

社会支持不足：缺乏亲密的、可信赖的人际关系，感到孤独和社会隔离。

重要理解：一个人患上抑郁症，不是他/她的错，也不是意志力薄弱的表现。风险因素只是增加了可能性，而保护性因素（如良好的社会支持、健康的应对技巧、积极的生活方式）则能提升心理韧性。识别这些因素，有助于我们理解抑郁症的复杂性，并转向寻求科学、有效的干预途径。

三、 寻求专业帮助是康复的第一步

抑郁症是一种可治疗、可管理的疾病。与高血压、糖尿病等慢性病类似，通过科学干预（如心理治疗、药物或两者结合），绝大多数患者的症状可以得到显著缓解，并恢复正常的社会功能。 如果您或您关心的人正经历上述困扰，鼓励您迈出关键一步：寻求精神科医生或临床心理师的专业评估。这并非软弱，而是对自己健康负责任和充满勇气的表现。 参考资料与权威来源：

世界卫生组织 (WHO). 《国际疾病分类》第11版 (ICD-11)。

美国精神医学学会 (APA). 《精神障碍诊断与统计手册》第5版 (DSM-5-TR)。

中国抑郁障碍防治指南。

美国国家心理卫生研究所 (NIMH) 官网公众科普信息。

**② Topic on diagnosis and assessment:**

Understanding the Diagnosis and Assessment of Depression: A Scientific and Empathic "Psychological Check-up"

When our body experiences persistent discomfort, we visit a physician. Similarly, when our emotional health has been "under the weather" for an extended period, a professional psychological check-up is called for. The diagnosis of depression is a systematic, rigorous, and empathic scientific process. Its purpose is not simply to assign a label but to gain a thorough understanding of the situation so that the most effective path to recovery can be mapped.

Core Principle: Professional Assessment Is the Foundation

Important note: Depression is a medical condition. It cannot be self-diagnosed through online quizzes or by checking a list of symptoms. A reliable diagnosis must be made by a trained psychiatrist or clinical psychologist. Like detectives, they piece together a complete picture through in-depth conversation and validated instruments.

What Does the Diagnostic Process Involve?

A typical professional assessment includes the following key components:

1. A thorough clinical interview: the cornerstone of diagnosis.

The clinician creates a safe, confidential environment in which to explore:

Your subjective experience: the nature of the low mood, the extent of anhedonia, and whether anxiety or irritability is present.

A comprehensive picture of daily life: changes in sleep, appetite, energy, concentration, and other physical and cognitive functions.

Duration: whether symptoms are present nearly every day and have persisted for at least two weeks.

Functional impact: the degree to which these symptoms interfere with work, studies, and relationships.

Other possible causes: the clinician carefully rules out physical conditions (such as thyroid disease) or substance effects that could account for the symptoms.

2. Standardized psychological assessment tools:

Clinicians may use internationally validated questionnaires, such as the Patient Health Questionnaire (PHQ-9), as supplementary references. These tools help quantify symptom severity, but they serve only as an aid to clinical judgment and can never replace it.

What Sets the Evaluation Apart: Beyond Symptoms to the Person

A professional evaluation goes beyond a symptom checklist. It also attends to:

Your individuality: the same depressive syndrome can manifest quite differently from one person to another. The assessment respects your unique personal history and background.

Comorbidity: the evaluation checks for co-occurring conditions, such as anxiety or substance use problems, because they influence the direction of treatment.

Risk and safety: the clinician carefully assesses any risk of self-harm or suicide. This is an essential part of the evaluation, conducted with genuine concern and aimed squarely at ensuring safety.

Seeking an Assessment Is a Courageous First Step

If you suspect you or someone close to you may be struggling with depression, actively seeking an assessment is the most important and courageous step toward recovery. It represents a decision to face and care for one's mental health.

You can contact the following resources for a professional evaluation:

Psychiatry or psychosomatic medicine departments in general hospitals

Specialized psychiatric hospitals

Professional psychological counselling centers

Scientifically Informed Self-Report Tools as a Reference

Before seeking professional help, if you wish to gain a preliminary sense of your emotional state, you may consider evidence-based self-report tools made available by authoritative bodies. These tools cannot diagnose, but they can help you describe your experience to a clinician more accurately.

For instance, the Mental Health Application Centre of the Institute of Psychology, Chinese Academy of Sciences, provides a platform with research-based psychological assessment instruments.

Please remember: the questionnaires on such platforms are tools for self-awareness and screening. The results must be interpreted and a diagnosis made by a qualified professional.

A final thought: arriving at a clear diagnosis means you can finally give a name to what has been troubling you and begin a targeted, supported journey of recovery. You are not alone, and scientifically grounded help is within reach.

Original Chinese:

理解抑郁症的诊断与评估：一次科学而温暖的“心理体检”

当我们的身体出现持续不适时，我们会去医院检查。同样，当我们的情绪长期“感冒”，也需要进行一次专业的“心理体检”。抑郁症的诊断正是一个严谨、系统且充满关怀的科学过程，其目的不是简单地贴标签，而是为了全面了解情况，从而找到最有效的康复路径。

核心原则：专业评估是基石

重要提示： 抑郁症是一种医学疾病，不能通过简单的网络自测题或对照症状清单来自行诊断。一个可靠的诊断必须由受过专业训练的精神科医生或临床心理师做出。他们会像侦探一样，通过深入的交谈和科学的工具，拼凑出完整的图像。

诊断过程是怎样的？

一次典型的专业评估通常包括以下几个关键部分：

1. 深入的临床访谈：这是诊断的核心。 医生会创造一个安全、保密的环境，与你探讨：

你的真实感受： 情绪低落的具体情况、兴趣丧失的程度、是否伴有焦虑或易怒。

全面的生活状态： 睡眠、食欲、精力、注意力等身体和思维上的变化。

持续的时间： 症状是否每天出现，并持续至少两周以上。

影响的程度： 这些症状对你的工作、学习、人际关系造成了多大的困扰。

其他可能原因： 医生会谨慎地排除其他可能导致类似症状的躯体疾病（如甲状腺问题）或药物影响。

2. 标准化的心理评估工具： 医生可能会使用一些国际公认的问卷（如PHQ-9，患者健康问卷-9）作为辅助参考。这些工具能帮助量化抑郁的严重程度，但它们只是医生的参考，绝不能替代医生的专业判断。

评估的关键：不止是症状，更是“人”

专业的评估不仅仅是核对症状清单，它更关注的是：

你的独特性： 同样的抑郁症状，在不同的人身上表现可能完全不同。评估会尊重你的个人经历和背景。

共病情况： 评估会检查是否同时存在焦虑、物质使用等其他问题，因为它们会影响治疗方向。

风险与安全： 医生会非常关切地评估任何自伤或自杀的风险，这是评估中至关重要的一环，目的是为了确保你的安全。

寻求评估是勇敢的第一步

如果你怀疑自己或亲友可能正受抑郁症困扰，主动寻求评估是迈向康复最重要、最勇敢的一步。这代表你开始正视并关心自己的心理健康。 您可以联系以下资源进行专业评估：

综合医院的精神科或心身医学科

精神专科医院

专业的心理咨询中心

科学自评工具作为参考

在寻求专业帮助前，如果你希望初步了解自己的情绪状态，可以参考一些权威机构发布的科学自评工具。这些工具不能用于自我诊断，但可以帮你更好地向医生描述你的情况。 例如，中国科学院心理研究所心理健康应用中心提供了一个专业的测评平台，其中包含基于科学研究的心理评估工具。 请记住： 平台上的问卷是帮助你觉察和筛查的工具，其结果必须由专业人士进行解读和诊断。 最后请理解： 获得一个清晰的诊断，意味着你终于可以为困扰你的问题命名，并开始一段有针对性的、充满支持的康复之旅。你并不孤单，科学的帮助触手可及。

**③ Topic on treatment and intervention:**

A Scientific Approach to Depression: Understanding Treatment and Moving Beyond Misconceptions

Receiving a diagnosis of depression can leave a person feeling bewildered, even frightened. In truth, depression—much like hypertension or diabetes—is a medical condition that is both treatable and manageable. Contemporary medicine has developed a range of evidence-based interventions. Familiarizing oneself with these methods, and clearing up the misunderstandings that so often surround them, constitutes a crucial step toward recovery.

I. Core Treatment Modalities: The Three Mainstays

Effective treatment generally requires a systematic plan. The three principal modalities outlined below are employed singly or in combination, depending on the clinician’s formulation of the individual case.

1. Psychotherapy (Talking Therapy)

This is not everyday conversation but a structured, goal-directed process guided by a trained therapist. Its central aim is to help the individual identify and modify the maladaptive patterns of thinking and behaving that precipitate or perpetuate depression, and to develop healthier coping strategies.

Common approaches: Cognitive behavioral therapy (CBT) and interpersonal psychotherapy (IPT), among others, have a strong evidence base for depression.

What it achieves: It alleviates current symptoms and, equally important, equips the person with psychological tools that guard against relapse.

2. Pharmacotherapy

Antidepressant medications act on the neurotransmitter systems implicated in mood regulation, helping to restore neurochemical balance and providing a physiological platform that supports psychotherapy and lifestyle change.

Essential points to grasp:

Not a “happy pill”: These agents do not manufacture artificial euphoria; they help recover the ordinary capacity to regulate mood.

Response takes time: Meaningful improvement typically requires two to four weeks, sometimes longer. Patience is indispensable.

Medical supervision is non-negotiable: The medication must be taken exactly as prescribed. Never alter the dose or stop on one’s own, as this can lead to poor response or a discontinuation syndrome.

3. Neuromodulation (Physical Therapies)

For moderate-to-severe depression, or when psychotherapy and medication have yielded an insufficient response, a number of safe neuromodulation approaches are available.

Electroconvulsive therapy (ECT): Performed under general anesthesia with muscle relaxation, modified ECT is a safe, highly effective treatment that can bring about rapid relief of severe symptoms. It is particularly indicated for life-threatening presentations or stupor. The public fear that often attaches to its older name is unwarranted; in modern practice it is a well-established therapeutic technique.

Repetitive transcranial magnetic stimulation (rTMS): This technique uses magnetic fields to stimulate targeted cortical regions non-invasively, without the need for anesthesia. It can be delivered on an outpatient basis.

Best practice increasingly follows a biopsychosocial integrative model: the above modalities are combined with lifestyle adjustments—regular exercise, attention to circadian rhythms and light exposure, strengthening of social support, and so forth—in a manner tailored to the individual.

II. Dispelling Myths and Fostering an Evidence-Based Outlook

Public misconceptions about the treatment of depression are often the single greatest barrier to seeking and adhering to care. Below, several common erroneous beliefs are examined and corrected.

Myth 1: “Medication will turn me into a zombie or get me hooked. Once I start, I’ll never be able to stop.”

What the evidence shows:

On “turning into a zombie”: Contemporary antidepressants are designed to improve, not impair, cognitive function. Depression itself causes difficulties with attention and memory—sometimes called “pseudodementia”—and as the medication lifts the depression, these cognitive symptoms generally lift as well. Some agents may produce initial side effects such as sedation, but these can be managed by working with the prescribing physician to adjust the regimen.

On “addiction”: Antidepressants do not produce the craving or euphoria that characterize substances of abuse, and they do not cause addiction. The body does develop physiological adaptation, and abrupt cessation can trigger transient symptoms such as dizziness or nausea—a discontinuation syndrome, which is not the same as withdrawal. The correct procedure is a gradual dose reduction over several weeks under medical guidance, allowing the brain to readapt smoothly.

Myth 2: “A troubled mind needs only talking. A chat with a friend should be enough; there is no need for medication.”

What the evidence shows: Depression arises from a confluence of biological, psychological, and social forces. In mild presentations, professional psychotherapy or lifestyle interventions alone may well be sufficient. In moderate-to-severe depression, however, neurochemical dysregulation has become a prominent physiological dimension of the illness. Just as a fracture requires a cast, medication serves as a necessary medical tool to correct that imbalance. Talking with a friend offers social support, which is valuable, but it is no substitute for professional psychotherapy or pharmacotherapy. Viewing treatment as addressing the physiological and the psychological in tandem reflects an evidence-based stance.

Myth 3: “Treatment means doing what the doctor says and taking the pills. I don’t have to do anything myself.”

What the evidence shows: Treatment is an active collaboration. The physician and therapist act as consultants and coaches; the individual is the principal agent of his or her own recovery. The heart of treatment is empowerment. Whether it is the skills acquired in psychotherapy or the window of emotional stability opened by medication, these must be actively applied in daily life—to adjust thinking, behavior, and lifestyle. The degree of one’s own engagement substantially influences both the pace and the extent of recovery.

Myth 4: “When I start feeling better, I can stop the medication myself. I’ll just take it again if things get worse.”

What the evidence shows: This is one of the most frequent causes of relapse. Symptom remission is merely the visible part of the iceberg that has melted; consolidation of underlying brain function takes considerably longer. Stopping medication prematurely is analogous to discontinuing an antibiotic before an infection has fully cleared—it markedly increases the likelihood that the illness will return, and subsequent treatment may become more complex. A complete course of treatment typically involves an acute phase (symptom control), a continuation phase (prevention of relapse), and for some individuals a maintenance phase (prevention of recurrence). Decisions about dose reduction or discontinuation must be made strictly on the basis of a professional assessment, in close consultation with the treating physician.

Myth 5: “A certain therapy or folk remedy worked wonders for someone else, so I should try it too.”

What the evidence shows: The treatment of depression must be individualized. Causative factors, symptom profiles, physical health status, and psychosocial context differ from person to person, so there is no single “best” treatment that suits everyone. Relying on unproven folk remedies—including the casual use of dietary supplements—can delay effective treatment and even produce dangerous interactions with prescribed medication. The most scientifically defensible course is to discuss the options thoroughly with one’s physician and co-create an evidence-based, personalized plan.

III. Moving Toward Recovery

It is worth stating clearly that seeking and adhering to treatment is a sign of strength, not weakness. The road to recovery is rarely a straight line, but every step counts. An open, trusting, collaborative relationship with one’s clinician is the bedrock of successful care. Should doubts or troubling side effects arise, they should be discussed with the physician promptly, rather than prompting a solitary decision. No one needs to grope in the dark alone. Scientifically grounded help, and genuine hope for recovery, are always within reach.

Original Chinese:

科学应对抑郁症：理解治疗，走出误区

当被诊断为抑郁症时，许多人会感到迷茫甚至恐惧。实际上，抑郁症如同高血压、糖尿病一样，是一种可治疗、可管理的医学疾病。现代医学已发展出多种经过科学验证的有效方法。了解这些方法，并澄清常见的误解，是走向康复的重要一步。

一、 核心治疗手段：三驾马车

抑郁症的有效治疗通常需要一个系统性的计划，主要分为三大类，医生会根据您的具体情况，建议单独或组合使用。

1. 心理治疗（谈话治疗） 这不是普通的聊天，而是一种有结构化目标、由受训治疗师引导的专业过程。其核心是帮助您识别并改变导致或加重抑郁的负面思维和行为模式，学习更健康的应对技巧。

常见方法：认知行为疗法、人际心理治疗等被证明对抑郁症尤其有效。

作用：不仅缓解当前症状，更能提供预防复发的心理工具。

2. 药物治疗 抗抑郁药物作用于大脑中与情绪调节相关的化学物质，帮助其恢复平衡，为心理治疗和生活调整提供“生理基础”。

关键认知：

并非“快乐丸”：它不创造虚幻的愉悦，而是帮助您恢复正常的情绪调节能力。

起效需要时间：通常需要2-4周甚至更久才能感受到明显改善，需要耐心。

需遵医嘱：必须在医生指导下使用，切勿自行增减或停药，以防效果不佳或引发撤药反应。

3. 物理治疗 对于中重度或对药物、心理治疗反应不佳的患者，有一些安全的物理方法。

无抽搐电休克治疗：在全身麻醉下进行，安全高效，能快速缓解严重症状，尤其对有生命危险或木僵状态的患者。公众因其旧称“电疗”而产生恐惧，实则是现代医学中一项成熟的治疗技术。

重复经颅磁刺激：利用磁场无痛地刺激大脑特定区域，无需麻醉，门诊即可进行。

最佳实践通常是“生物-心理-社会”综合模式，即根据个体情况，将上述方法与生活方式调整（规律运动、光照、社会支持等）相结合。

二、 澄清误区，树立科学信念

公众对抑郁症治疗的误解，常常成为寻求和坚持治疗的最大障碍。以下是一些常见错误信念的详细修正：

误区1：“吃药会让人变傻/上瘾，一旦吃上就停不掉了。”

科学修正：

1. 关于“变傻”：现代抗抑郁药物旨在改善认知功能。抑郁症本身就会导致注意力、记忆力下降（“假性痴呆”），药物在缓解抑郁的同时，通常能改善这些认知症状。部分药物在初期可能有嗜睡等副作用，但可通过与医生沟通调整来管理。

2. 关于“上瘾”：抗抑郁药不会像毒品或镇静剂那样引起渴求感和欣快感，因此不具有“成瘾性”。但身体可能会产生“适应”，突然停药可能出现头晕、恶心等“撤药反应”，这不同于“戒断症状”。正确做法是在医生指导下，用数周时间缓慢减量直至停药，让大脑平稳适应。

误区2：“心病只需心药医，找朋友聊聊天就行，不用吃药。”

科学修正： 抑郁症是生物、心理、社会因素共同作用的结果。对于轻度抑郁，专业的心理治疗或生活方式干预可能足够。但对于中重度抑郁，大脑的神经化学物质失衡已成为一个突出的生理问题，如同骨折需要石膏固定一样，药物是纠正这种失衡的重要医学工具。“朋友聊天”提供的是社会支持，无法替代专业的心理治疗或药物治疗。将治疗视为“生理与心理双管齐下”，才是科学的态度。

误区3：“治疗就是听医生的话吃药，自己不用做什么。”

科学修正： 治疗是一个主动合作的“团队工作”，医生和治疗师是您的顾问和教练，而您自己才是康复的主体。治疗的核心是授人以渔。无论是心理治疗中学到的技巧，还是药物带来的情绪稳定窗口期，都需要您主动地应用于生活，调整思维、行为和生活方式。您的主动参与程度，极大地影响着康复的效果和速度。

误区4：“感觉好点了就可以自己停药，不舒服再吃。”

科学修正： 这是导致复发的最常见原因之一。症状缓解只是“冰山一角”消融，大脑功能的巩固需要更长时间。过早停药如同在感染未愈时停用抗生素，极易导致病情反复，且后续治疗可能更复杂。全程治疗通常分为急性期（消除症状）、巩固期（防止复发）和维持期（预防再发），何时减量、停药，必须严格遵循医嘱，基于对您病情的专业评估。

误区5：“某种疗法/偏方对别人特别有效，我也要尝试。”

科学修正： 抑郁症的治疗强调“个体化”。每个人的病因、症状组合、身体状况、社会背景都不同，因此不存在“对所有人都最好的”单一疗法。迷信偏方（如随意服用补充剂）可能延误治疗，甚至与药物发生危险相互作用。最科学的路径，是与您的医生充分沟通，基于证据共同制定适合您的个性化方案。

三、 迈向康复之路

请记住，寻求和坚持治疗是力量的象征。康复之路可能并非一帆风顺，但每一步都算数。与您的医生建立开放、信任的合作关系，是成功治疗的基础。 如果您对治疗有疑虑或副作用困扰，及时与医生讨论，而不是独自决定。您不必独自在黑暗中摸索，科学的帮助和真正的康复希望，一直存在。

**④ Topic on prevention and self-care:**

Safeguarding the Mind's Oasis: Prevention and Self-Care in Depression

At its core, the prevention of depression centers on proactively strengthening our psychological immune system and building a resilient inner barrier through evidence-informed lifestyle practices and cognitive habits. While it is impossible to guarantee absolute immunity, active self-care substantially lowers risk and enhances overall quality of life. This is not a demand to feel happy at all times; rather, it is about learning to build buffers into daily living so that we can navigate stress and adversity more effectively.

I. Scientifically Informed Prevention: Constructing a Three-Tier Protective Net

Effective prevention is a systematic undertaking. It can be approached at three levels.

1. Foundational protection: lifestyle cornerstones for everyone

Stabilizing daily rhythms: Maintain a broadly regular sleep–wake schedule, aiming for 7–9 hours of good-quality sleep. Eat at regular intervals and attend to balanced nutrition.

Physical activity, a natural remedy: Engage in at least 150 minutes of moderate-intensity exercise each week (e.g., brisk walking, cycling). Exercise naturally modulates the brain chemistry implicated in mood regulation.

Stress management and timely unwinding: Learn to recognize one’s own stress signals and establish everyday relaxation rituals, such as mindful breathing, brief meditation, or a quiet walk.

2. Early identification: attending to high-risk phases and signals

Attending to critical junctures: Following major bereavement, significant role transitions (e.g., postpartum, retirement), the onset of chronic illness, or prolonged periods of high stress, make a deliberate effort to increase self-care.

Recognizing warning signs: When a low mood or markedly diminished interest persists for more than two weeks and is accompanied by notable changes in sleep and appetite, regard it as a psychological alarm that calls for proactive intervention.

Seeking assessment proactively: Individuals with a family history of depression, or anyone who senses that their mood has been persistently poor, may consider a professional mental health evaluation, much as one would schedule a routine physical check-up.

3. Consolidation and maintenance: preventing recurrence as a key objective

Completing the full course of treatment: For those who have experienced an episode, adhering to the full, clinically prescribed course of treatment is the single most important measure to prevent recurrence.

Recognizing personal patterns: Become familiar with one’s own early warning signs of recurrence (e.g., the reappearance of specific negative thoughts, social withdrawal) and develop a coping plan to address them.

Sustained application of skills: Transform the cognitive and behavioral regulation techniques learned during treatment into enduring lifestyle habits.

II. Self-Care: Practical Everyday Actions

Self-care is the active practice of attending to one’s own mental health; it is not self-indulgence. It encompasses the following dimensions.

1. Emotional and cognitive self-care

Making room for emotions: Acknowledge and accept the validity of all emotional states—painful ones included—without judgement.

Balancing automatic thoughts: When caught in all-or-nothing thinking, make a deliberate effort to search for alternative possibilities or disconfirming evidence.

Noticing positive experiences: Each day, consciously attend to and jot down a few small moments that bring a glimmer of positivity, cultivating an attitude of gratitude.

2. Relationships and social connection

Maintaining core supports: Proactively nurture at least one or two trusting relationships that allow for in-depth conversation and mutual support.

Engaging in community: Take part in group activities aligned with one’s interests to foster a sense of belonging and a meaningful social role.

Practicing effective communication: Learn to express needs and feelings, and, when necessary, set interpersonal boundaries with gentleness.

3. Physical and environmental regulation

Curating one’s digital life: Manage screen time intentionally, especially before bed, to reduce the stress associated with information overload and social comparison.

Creating restorative space: Carve out a physical environment in which one feels safe and can genuinely unwind.

Contact with the natural environment: Schedule regular time in natural light and green surroundings.

III. Important Reminders: A Realistic Perspective on Prevention

Prevention is not elimination: The goal is to build resilience, not to eradicate all stress or low mood—an unrealistic expectation.

Self-care is not selfishness: Looking after oneself is the very foundation from which one can care for others and fulfil responsibilities effectively.

Help-seeking is an essential competence: When one’s own coping efforts prove insufficient, actively seeking professional help is a mark of wisdom and strength.

Health is a dynamic process: Mental health requires ongoing maintenance, just as physical health requires balanced nutrition and exercise.

Summary Points: Stable routines anchor body and mind; movement and connection form the bedrock. Notice warning signals early and respond; learn skills for active regulation. Sustained self-care thrives on commitment; when difficulties arise, turn to professionals.

Original Chinese:

守护心灵绿洲：抑郁症的预防与自我关怀

抑郁症的预防，核心在于通过科学的生活方式与认知习惯，主动提升我们的心理免疫力，构建坚韧的内心屏障。虽然无法保证绝对不患病，但积极的自我关怀能显著降低风险，并改善整体生活质量。这并非要求我们时刻保持快乐，而是学会在生活中建立缓冲，更好地应对压力与逆境。

一、 科学预防：构建三层防护网

有效的预防是一个系统工程，可从三个层面着手：

1. 基础防护：面向所有人的生活方式基石

规律节律，稳定身心：保持每天大致固定的睡眠与起床时间，优先保证7-9小时优质睡眠。规律进食，注重均衡营养。

身体活动，天然良药：每周坚持至少150分钟中等强度运动（如快走、骑行）。运动能自然调节大脑内与情绪相关的化学物质。

压力管理，及时松绑：学习识别自己的压力信号，并建立日常放松仪式，如正念呼吸、短暂冥想或散步。

2. 早期识别：关注高风险阶段与信号

关注关键阶段：在经历重大丧失、角色转变（如产后、退休）、慢性疾病或长期高压后，需有意识增加自我关照。

识别预警信号：当情绪低落、兴趣减退持续超过两周，并伴有睡眠、食欲明显变化时，应视作需要主动干预的“心理警报”。

主动寻求评估：若有抑郁症家族史，或自觉情绪持续不佳，可像定期体检一样，考虑进行专业的心理健康评估。

3. 巩固维持：预防复发是关键环节

完成全程治疗：若已患病，遵医嘱完成足疗程治疗是预防复发的最重要措施。

识别个人模式：了解自己复发的早期征兆（如特定负面想法重现、社交退缩），并制定应对计划。

持续应用技能：将在治疗中学到的认知与行为调节技巧，转化为长期的生活习惯。

二、 自我关怀：可实践的日常行动

自我关怀是主动照顾自己心理健康的实践，而非自我放纵。它包含以下维度：

1. 情绪与思维关怀

允许情绪存在：承认并接纳所有情绪（包括痛苦情绪）的合理性，不加以批判。

平衡自动思维：当陷入“全或无”的极端想法时，尝试寻找其他可能性或证据。

记录积极体验：每天有意识地关注并记录几件带来微小积极感受的事，培育感恩心态。

2. 关系与社会连接

维护核心支持：主动维系至少1-2段能深度交谈、给予支持的信任关系。

参与社群活动：根据兴趣参与团体活动，建立归属感与社会角色。

练习有效沟通：学会表达需求与感受，并在必要时温和地设立人际边界。

3. 身体与环境调节

优化数字生活：有意识地管理屏幕时间，特别是在睡前，减少信息过载与社交比较带来的压力。

创造恢复空间：为自己营造一个能感到安全、放松的物理环境。

接触自然环境：定期安排时间接触自然光线与绿色环境。

三、 重要提醒：关于预防的理性认知

预防不等于杜绝：目标是提升韧性，而非消除所有压力与低落情绪，那是不现实的。

自我关怀非自私：照顾好自己是能够有效关心他人和履行责任的基础。

求助是必要能力：当自我调节效果有限时，主动寻求专业帮助是智慧与力量的体现。

健康是动态过程：心理健康需持续维护，如同身体健康需要均衡饮食与锻炼。

总结要点 规律作息稳身心， 运动社交是基石。 觉察信号早应对， 主动调节学技巧。 持续关怀贵坚持， 遇有困难寻专业。

**⑤ Topic on social support and recovery:**

Social Support in Depression Recovery: Evidence-Based Communication and Effective Companionship

The journey of recovery from depression is not travelled alone. Social support is a critical factor shaping the course of recovery. The World Health Organization has repeatedly emphasized that family and community support constitute an essential pillar of mental health care. Informed, evidence-based support can not only alleviate the sense of stigma but also enhance treatment adherence and overall recovery outcomes.

I. Core Principles of Effective Support

1. Listening takes precedence over advising.

Research indicates that non-judgmental listening reduces emotional distress in the person with depression. Supporters should resist the urge to offer premature advice, such as “just try to think positively,” and instead employ empathic statements like “I’m here with you” or “That must feel truly difficult,” conveying genuine acceptance.

2. Respect the nature of the illness.

Diminished interest, irritability, and social withdrawal are symptoms of depression, not character flaws. The American Psychiatric Association has noted that pressuring a person to “pull themselves together” can intensify feelings of self-blame. It is more helpful to allow the individual to recover at a pace they can manage.

3. Offer concrete, actionable support.

Rather than making a vague offer (“Let me know if you need anything”), it is more effective to provide specific, manageable assistance—for instance, “I can help you book the appointment with the doctor” or “How about I join you for a half-hour walk this weekend?” Empirical studies show that practical action alleviates feelings of helplessness more than verbal reassurance alone.

II. Common Misunderstandings and Evidence-Based Corrections

Misunderstanding 1: “Talking about depression too often will only make the person feel worse.”

Evidence-based correction: Research demonstrates that expressing emotion in a safe environment facilitates emotional regulation. Avoiding the topic altogether can leave the person feeling isolated and unsupported. The appropriate approach is to gently ask, “Would you like to talk about how you’re feeling? It’s perfectly fine if you’d rather not,” thereby giving the person full agency over disclosure.

Misunderstanding 2: “Being supportive means keeping a constant watch on the person to prevent anything untoward.”

Evidence-based correction: Over-monitoring can generate a sense of suffocation. High-quality companionship involves maintaining a state of availability—checking in by message at intervals, for example—while respecting the person’s need for solitude. Crisis preparedness should focus primarily on connecting the person to professional resources, such as accompanying them to an appointment or keeping a helpline number readily accessible.

Misunderstanding 3: “Being supportive means cheering the person up and making them happy again.”

Evidence-based correction: Depression is a condition with a neurobiological substrate; positive mood cannot simply be forced. Pressuring the person to engage in cheerful activities can inadvertently heighten distress. A more evidence-informed approach is to accept the low mood while providing low-demand companionship, such as listening to music together or sharing a quiet meal.

III. Self-Care for the Supporter

Supporters must guard against becoming overly enmeshed. The Chinese Psychological Society recommends applying the “airplane oxygen mask principle”: secure your own emotional stability first. Set clear boundaries around the support you provide—for example, by confining in-depth conversations to an agreed-upon period of the day—and seek guidance from a mental health professional when necessary.

Summary Points: Listen without judging, accompany without pushing. Let actions speak louder than words, with professional help as the backstop. Maintain clear boundaries, and protect yourself first.

Original Chinese:

抑郁症康复中的社会支持：科学沟通与有效陪伴

抑郁症的康复之路并非独行，社会支持是影响康复进程的关键因素。世界卫生组织多次强调，家庭与社会支持是心理健康服务的重要支柱。科学的支持不仅能减轻患者的病耻感，更能提升治疗依从性与康复效果。

一、有效支持的核心原则

1. 倾听优于指导 研究发现，不加评判的倾听能降低患者的情绪压力。支持者需避免急于给出建议（如“你想开点就好了”），而应使用“我在这里陪你”“这种感觉一定很难受”等共情语言，传递接纳感。

2. 尊重疾病规律 抑郁症患者常出现兴趣减退、易怒或社交回避，这些是症状而非性格缺陷。美国精神医学学会指出，强迫患者“振作”可能加剧其自责感，应允许对方按自身节奏恢复。

3. 提供具体支持 比起笼统的“需要帮忙随时说”，更有效的做法是主动提供可执行帮助，如“我帮你预约医生”“周末陪你散步半小时”。实证研究显示，实际行动比语言安慰更能缓解患者的无助感。

二、常见误解与科学修正

误解1：“经常谈论抑郁会加重患者情绪。” 科学修正： 研究表明，安全的环境下表达情绪有助于情绪调节。回避谈论反而会让患者感到孤独。正确做法是温和询问“你想聊聊感受吗？不想说也没关系”，给予表达主动权。

误解2：“陪伴就是时刻盯着患者防止意外。” 科学修正： 过度监控可能引发窒息感。高质量陪伴是“保持可及性”，如定期发消息问候，同时尊重对方独处需求。危机干预重点应放在专业资源对接上（如陪同就诊、存好心理热线）。

误解3：“支持就是让患者开心起来。” 科学修正： 抑郁症是生理性病症，正面情绪难以强制激发。强行组织娱乐活动可能增加患者压力。更科学的方式是接纳低落情绪，陪伴进行低压力活动（如一起听音乐、安静用餐）。

三、支持者的自我防护

支持者需避免过度卷入。中国心理学会建议采用“飞机氧气面罩原则”：先保障自身情绪稳定，设置支持边界（如每天固定时间段沟通），必要时寻求心理咨询师指导。

总结要点 倾听不评判，陪伴不催促；行动胜语言，专业做后盾；边界需明确，自我保护先。

**(2) The baseline prompt group:**

**① Topic on causes and risk factors:**

Depression: Understanding, Recognizing, and Facing It

What Is Depression?

Depression, also referred to as depressive disorder, is a common yet serious mental health condition. It is neither simply “feeling down” nor a passing episode of low mood, but a medical condition that warrants professional intervention. Depression affects how a person thinks, feels, and functions, significantly interfering with daily life, work, and interpersonal relationships. It is not a character weakness or an emotional state one can “snap out of,” but a complex biopsychosocial syndrome that affects hundreds of millions of people worldwide.

Core Symptoms

According to international diagnostic criteria, the core symptoms of depression include the following:

Core affective symptoms:

Persistent low mood: feeling sad, empty, or hopeless for most of the day, nearly every day.

Markedly diminished interest or pleasure: losing interest in almost all activities, including those one used to enjoy.

Other common symptoms:

Significant weight change or change in appetite.

Sleep disturbance (insomnia or hypersomnia).

Psychomotor agitation or retardation.

Fatigue or loss of energy.

Feelings of worthlessness or excessive or inappropriate guilt.

Diminished ability to think or concentrate, or indecisiveness.

Recurrent thoughts of death, suicidal ideation, or a suicide attempt.

A diagnosis typically requires that at least five of the above symptoms (which must include at least one core affective symptom) be present during the same two-week period and represent a change from previous functioning, while causing clinically significant distress or functional impairment.

Causes and Risk Factors

The etiology of depression is complex and generally understood as the product of an interplay among biological, psychological, and social factors.

Biological factors:

Alterations in brain chemistry, particularly involving neurotransmitters such as serotonin, norepinephrine, and dopamine.

Genetic liability (a family history raises risk but is not a deterministic factor).

Hormonal changes.

Chronic physical illness or persistent pain.

Psychological factors:

Negative cognitive patterns and habitual thinking styles.

Low self-esteem.

Childhood trauma or neglect.

Certain personality traits.

Social and environmental factors:

Major life events (bereavement, unemployment, relationship breakdown).

Chronic stress.

Social isolation or a weak support system.

Socioeconomic adversity.

Certain medications or substance misuse.

Important Note

Depression is a treatable condition. Effective treatments include psychotherapy (such as cognitive behavioral therapy), pharmacotherapy (such as antidepressant medication), or a combination of the two. Lifestyle adjustments, social support, and stress management are also beneficial to recovery.

If you or someone you know may be experiencing depression:

Seeking professional help is the first step toward recovery.

Depression is neither a personal failure nor a character flaw.

Early intervention generally leads to better recovery outcomes.

A supportive environment that reduces stigma is critical to the healing process.

Please remember that mental health is as important as physical health, and seeking help is an act of strength and courage. If you are in crisis, please contact a local mental health helpline or seek immediate medical attention.

Original Chinese:

抑郁症：理解、识别与面对

什么是抑郁症？

抑郁症（又称抑郁障碍）是一种常见但严重的心理健康疾病，它不仅仅是“心情不好”或“暂时的情绪低落”，而是一种需要专业干预的医学状况。抑郁症会影响一个人的思考、感受和行为，显著干扰日常生活、工作和人际关系。 抑郁症不是性格弱点或可以“简单摆脱”的情绪状态，而是一种复杂的生物心理社会综合症，影响着全球数亿人。

核心症状

根据国际诊断标准，抑郁症的核心症状包括： 核心情绪症状：

持续的情绪低落：几乎整天、每天感到悲伤、空虚或无望

兴趣或愉悦感丧失：对几乎所有活动失去兴趣，包括曾经喜欢的活动

其他常见症状：

显著的体重变化或食欲改变

睡眠障碍（失眠或过度睡眠）

精神运动性激越或迟滞

疲劳或精力丧失

无价值感或过度或不恰当的内疚

思考或专注能力下降，犹豫不决

反复出现的死亡念头、自杀意念或自杀尝试

诊断通常要求上述症状中至少5项（必须包括至少一项核心情绪症状），持续至少两周，并导致显著的功能损害。

成因与风险因素

抑郁症的成因复杂，通常是生物、心理和社会因素相互作用的结果：

生物因素：

大脑化学物质失衡，特别是神经递质如血清素、去甲肾上腺素和多巴胺

遗传倾向（家族史会增加风险，但并非绝对决定因素）

荷尔蒙变化

慢性疾病或疼痛

心理因素：

消极的认知模式和思维习惯

低自尊

童年创伤或忽视

特定的性格特质

社会与环境因素：

重大生活事件（失去亲人、失业、关系破裂）

长期压力

社会孤立或支持系统薄弱

社会经济压力

某些药物或物质滥用

重要提示

抑郁症是可治疗的疾病。有效的治疗方法包括心理治疗（如认知行为疗法）、药物治疗（如抗抑郁药）、或两者结合。生活方式调整、社会支持和压力管理也有助于康复。 如果您或您认识的人可能正在经历抑郁症：

寻求专业帮助是康复的第一步

抑郁症不是个人的失败，也不是性格缺陷

早期干预通常能带来更好的恢复效果

提供支持、减少病耻感的环境对康复至关重要

请记住，心理健康与身体健康同样重要，寻求帮助是力量和勇气的表现。如果您正经历危机，请联系当地心理健康热线或立即就医。

**② Topic on diagnosis and assessment:**

Diagnosis and Assessment of Depression: Scientific Identification and Professional Support

Diagnostic Principles: Professional, Systematic, and Multidimensional

The diagnosis of depression is a rigorous clinical process and should not be attempted through self-rating scales or online quizzes. An accurate diagnosis must be made by a psychiatrist, a clinical psychologist, or another qualified mental health professional and typically involves the following key components.

1. Clinical Interview and Evaluation

Through a structured interview, the professional will explore:

Symptom characteristics: their nature, severity, and duration.

Functional impact: the degree to which they interfere with work, study, and interpersonal relationships.

Time criterion: whether the symptoms have persisted for at least two weeks.

Exclusion of other causes: ensuring the symptoms are not attributable to another medical condition, medication, or substance.

2. Standardized Diagnostic Instruments

Professionals may employ:

Clinician-rated scales: such as the Hamilton Depression Rating Scale (HAM-D) or the Montgomery-Åsberg Depression Rating Scale (MADRS).

Structured interview tools: such as the Mini-International Neuropsychiatric Interview (M.I.N.I.).

Self-report measures as adjuncts: such as the Patient Health Questionnaire (PHQ-9), though these serve only as references and cannot replace clinical diagnosis.

Core Dimensions of the Assessment

Symptom Assessment

Affective symptoms: the quality, intensity, and diurnal or situational variation of the depressed mood.

Somatic symptoms: changes in sleep, appetite, energy, and the presence of bodily pain.

Cognitive symptoms: alterations in attention, memory, and decision-making capacity.

Behavioral symptoms: social withdrawal and changes in activity level.

Functional Assessment

Extent of impairment in everyday functioning.

Capacity to fulfil social roles.

Impact on quality of life.

Risk Assessment

Suicide risk: ideation, plans, intent, and past behavior.

Self-harm risk.

Self-care capacity: assessment of basic ability to look after oneself.

Differential Diagnosis: Ruling Out Similar Presentations

The clinician carefully distinguishes depression from:

Other mental disorders: bipolar disorder, anxiety disorders, and adjustment disorders.

Depressive presentations due to physical illness: such as thyroid disease or neurological conditions.

Substance- or medication-induced depressive symptoms.

Normal bereavement responses.

Special Considerations in the Assessment

Differences Across Populations

Children and adolescents: may present with irritability rather than a plainly depressed mood.

Older adults: may emphasize somatic complaints, which can mask an underlying affective disturbance.

Varied cultural backgrounds: the expression and subjective experience of depression can differ across cultures.

Comorbidity Assessment

Approximately two-thirds of individuals with depression have a co-occurring mental disorder.

Common comorbidities include anxiety disorders, substance use disorders, and personality disorders.

Chronic physical illness and depression often exert a reciprocal influence on one another.

Pathways to Seeking an Assessment

1. Primary care settings: general practitioners can perform initial screening and make appropriate referrals.

2. Specialized psychiatric hospitals: provide comprehensive evaluation and diagnosis.

3. Psychiatry or psychosomatic medicine departments in general hospitals.

4. Community mental health service centers.

Important Reminders

Diagnosis is a process: it may require more than one interview and period of observation.

Honest communication is essential: describing one’s symptoms and experiences fully and truthfully is paramount.

Diagnosis is the beginning of help: obtaining a clear diagnosis means that targeted, effective treatment can commence.

A diagnostic label does not define the person: depression is a condition one experiences; it is not the entirety of one’s identity.

If you suspect that you or someone you care about may be experiencing depression, seeking a professional assessment is the crucial first step. Early identification and intervention significantly improve prognosis. Mental health is every bit as important as physical health and merits the same degree of attention and professional care.

Original Chinese:

抑郁症诊断与评估：科学识别与专业支持

诊断原则：专业、系统、多维

抑郁症的诊断是一个严谨的临床过程，不应通过自评量表或网络测试自行诊断。正确的诊断需要由精神科医生、心理医生或其他有资质的心理健康专业人员完成，通常包括以下几个关键环节：

1. 临床面谈与评估

专业人员会通过结构化的访谈了解：

症状特征：类型、严重程度、持续时间

功能影响：对工作、学习、人际关系的影响程度

时间标准：症状是否持续至少两周

排除其他可能：确保症状不是由其他疾病、药物或物质引起

2. 标准化的诊断工具

专业人员可能使用：

临床评定量表：如汉密尔顿抑郁量表、蒙哥马利抑郁量表

结构化访谈工具：如迷你国际神经精神访谈

自评量表辅助：如患者健康问卷，但这些仅作为参考，不能替代临床诊断

评估的核心维度

症状评估

情绪症状：抑郁情绪的性质、强度、波动规律

躯体症状：睡眠、食欲、精力、疼痛等身体变化

认知症状：注意力、记忆力、决策能力变化

行为症状：社交退缩、活动水平变化

功能评估

日常功能受损程度

社会角色履行能力

生活质量影响

风险评估

自杀风险：想法、计划、意图、既往行为

自伤风险

自理能力：基本自我照顾能力评估

鉴别诊断：排除类似情况

医生会谨慎区分抑郁症与：

其他精神障碍：双相情感障碍、焦虑障碍、适应障碍

躯体疾病所致抑郁：甲状腺疾病、神经系统疾病等

物质或药物引起的抑郁症状

正常的哀伤反应

评估中的特殊考虑

不同人群的差异

儿童青少年：可能表现为易怒而非情绪低落

老年人：可能强调躯体症状，掩盖情绪问题

不同文化背景：抑郁的表达和认知可能存在文化差异

共病评估

约2/3的抑郁症患者同时患有其他精神障碍

常见共病：焦虑障碍、物质使用障碍、人格障碍

慢性躯体疾病与抑郁常相互影响

寻求评估的途径

1. 初级医疗机构：全科医生可进行初步筛查和转介

2. 精神专科医院：提供全面评估和诊断

3. 综合医院精神科或心身医学科

4. 社区心理健康服务中心

重要提醒

诊断是一个过程：可能需要多次访谈和观察

诚实沟通至关重要：完整、真实地描述症状和经历

诊断是帮助的开始：获得明确诊断意味着可以开始针对性的有效治疗

诊断标签不是定义：抑郁症是您经历的状况，不是您的全部身份

如果您怀疑自己或关心的人可能有抑郁症，寻求专业评估是至关重要的第一步。早期识别和干预能显著改善预后。心理健康与身体健康同样重要，值得同等的关注和专业照护。

**③ Topic on treatment and intervention:**

Treatment and Intervention for Depression: Evidence-Based Approaches and the Road to Recovery

Core Principle: Individualized and Integrated Treatment

Depression is a treatable medical condition. Effective intervention typically requires a systematic approach delivered under professional guidance. Modern psychiatry has developed a range of evidence-based treatments for depression, and the substantial majority of individuals who receive appropriate care achieve clinically meaningful improvement.

Principal Treatment Modalities

1. Psychotherapy (Talking Therapy)

Cognitive behavioral therapy (CBT)

Assists in identifying and modifying maladaptive thought patterns and behaviors.

Teaches coping skills to manage depressive symptoms.

Particularly efficacious for mild-to-moderate depression.

Interpersonal psychotherapy (IPT)

Focuses on the role of interpersonal difficulties in depression.

Improves communication skills and social functioning.

Especially effective when depression has been precipitated by interpersonal stress.

Other empirically supported approaches: psychodynamic psychotherapy, mindfulness-based cognitive therapy (MBCT), and acceptance and commitment therapy (ACT).

2. Pharmacotherapy

Principal classes:

Selective serotonin reuptake inhibitors (SSRIs): widely used as first-line agents.

Serotonin-norepinephrine reuptake inhibitors (SNRIs).

Antidepressants with other mechanisms of action.

Essential considerations:

Therapeutic response typically requires 2–4 weeks to become evident.

The full course of treatment must be completed as prescribed; self-initiated discontinuation should be avoided.

Regular follow-up appointments are necessary to adjust dosing and manage side effects.

The goal of treatment is generally full remission, not merely partial improvement.

3. Neuromodulation (Physical Therapies)

Repetitive transcranial magnetic stimulation (rTMS): indicated when the response to medication has been insufficient.

Electroconvulsive therapy (ECT): a highly effective and safe intervention for severe depression, high suicide risk, or pharmacotherapy-resistant cases.

Vagus nerve stimulation (VNS): considered in chronic, treatment-resistant depression.

Integrated Care: A Multi-Layered Support System

Lifestyle Interventions

Regular exercise: weekly moderate aerobic activity has a well-documented antidepressant effect.

Stable daily rhythms: maintaining a consistent sleep–wake cycle supports mood regulation.

Nutritional support: a balanced diet, with attention to nutrients implicated in mood.

Light therapy: particularly useful for seasonal depression.

Social Support

Family understanding and involvement can measurably improve treatment outcomes.

Support groups offer a sense of belonging and the opportunity to share lived experience.

Reasonable adjustments in the workplace can sustain the recovery process.

Phases of Treatment and Their Goals

Acute phase (6–12 weeks)

Goal: eliminate the principal symptoms and restore basic functioning.

Approach: psychotherapy, pharmacotherapy, or a combination of the two.

Continuation phase (4–9 months)

Goal: prevent relapse and sustain the gains achieved during the acute phase.

Approach: continue the treatment regimen that proved effective.

Maintenance phase (1 year or longer)

Goal: prevent recurrence, especially in individuals at elevated risk.

Approach: treatment may be continued or gradually tapered, depending on clinical judgment.

Special Considerations for Specific Populations

Adolescents: psychotherapy is often the first-line intervention; pharmacotherapy requires particular caution.

Pregnancy and the postpartum period: the health of the mother must be weighed carefully against potential risks to the fetus or infant.

Older adults: attention to drug–drug interactions and co-existing medical conditions is essential.

Individuals with comorbidity: depression alongside other mental or physical disorders requires integrated, coordinated care.

Practical Steps for Seeking Help

1. Professional assessment: a psychiatrist or clinical psychologist conducts a comprehensive evaluation.

2. Shared decision-making: treatment options are discussed collaboratively with the clinician.

3. Initiation of treatment: the agreed-upon plan is put into action.

4. Periodic review: progress is monitored and the treatment strategy is adjusted accordingly.

5. Recovery management: skills to prevent relapse and recurrence are developed.

Important Reminders

Treatment takes time: improvement in depressive symptoms is typically gradual.

Effective alternatives exist: if one approach yields an insufficient response, several evidence-based options remain.

Treatment adherence is critical: discontinuing treatment prematurely is one of the principal causes of relapse.

Recovery is attainable: 70–80% of individuals show a favorable response to appropriate treatment.

If you are living with depression, seeking professional help is the first step toward recovery. The process may require patience and persistence, but with proper intervention, most people regain a meaningful level of functioning and life satisfaction. The road to recovery need not be walked alone; professional support and social networks can both provide the assistance that is necessary.

Original Chinese:

抑郁症治疗与干预：科学方法与康复之路

核心原则：个体化综合治疗

抑郁症是一种可治疗的医学疾病，有效治疗通常需要专业指导下的系统干预。现代医学针对抑郁症已发展出多种循证治疗方法，其中大部分患者通过适当治疗可获得显著改善。

主要治疗方法

1. 心理治疗（谈话治疗）

认知行为疗法

帮助识别和改变负面思维模式与行为

教授应对技巧应对抑郁症状

尤其对轻中度抑郁有效

人际治疗

关注人际关系在抑郁中的作用

改善沟通技巧和社交功能

对由人际压力引发的抑郁特别有效

其他有效疗法：心理动力学治疗、正念认知疗法、接纳与承诺治疗

2. 药物治疗

主要类别：

选择性5-羟色胺再摄取抑制剂：常见一线药物

5-羟色胺和去甲肾上腺素再摄取抑制剂

其他机制抗抑郁药

关键须知：

药物通常需要2-4周开始显现效果

需遵医嘱完成整个疗程，避免自行停药

定期复诊以调整剂量和管理副作用

治疗目标通常是完全缓解而非部分改善

3. 物理治疗

重复经颅磁刺激：适用于药物疗效有限者

电抽搐治疗：对严重抑郁、有自杀风险或药物治疗无效者高效且安全

迷走神经刺激：适用于慢性难治性抑郁

整合治疗：多层次支持系统

生活方式干预

规律运动：每周适度有氧运动有明确抗抑郁效果

规律作息：稳定睡眠-觉醒节律有助于情绪调节

营养支持：均衡饮食，关注与情绪相关的营养素

光照疗法：尤其对季节性抑郁有效

社会支持

家庭理解和参与可显著改善治疗结果

支持性团体提供归属感和经验分享

工作场所的合理调整支持康复过程

治疗阶段与目标

急性期（6-12周）

目标：消除主要症状，恢复基本功能

方法：心理治疗、药物治疗或二者结合

巩固期（4-9个月）

目标：防止复发，维持已获得的改善

方法：继续有效治疗方案

维持期（1年或更久）

目标：预防新发，特别是对复发风险高者

方法：可能继续或逐渐减少治疗强度

特殊人群治疗考量

青少年：心理治疗常为首选，药物使用需谨慎

孕期和哺乳期：需平衡母体健康与胎儿安全

老年人：注意药物相互作用和躯体共病

共病患者：需综合治疗抑郁症与其他精神或躯体疾病

寻求帮助的实用步骤

专业评估：精神科医生或心理医生进行全面评估

共同决策：与专业人员讨论治疗方案选择

开始治疗：按计划执行治疗方案

定期评估：监控进展，调整治疗策略

康复管理：学习预防复发技能

重要提醒

治疗需要时间：抑郁症状改善通常是渐进的

存在有效选择：如果一种方法效果不足，有多种备选方案

治疗依从性至关重要：中断治疗是复发的主要原因之一

康复是可能的：70-80%患者对适当治疗有良好反应

如果您正在经历抑郁，寻求专业帮助是走向康复的第一步。治疗过程可能需要耐心和坚持，但多数人通过适当干预能够重新获得生活的满足感和功能水平。恢复之路不必独自前行，专业支持和社交网络都能为您提供必要帮助。

**④ Topic on prevention and self-care:**

The Prevention of Depression and the Practice of Self-Care: Daily Habits That Safeguard Mental Health

Understanding Prevention: A Multi-Layered, Practical Protective Framework

The onset of depression involves an interplay of biological, psychological, and social factors. While no approach can provide absolute protection, evidence-informed lifestyle practices and cognitive habits can substantially lower risk and strengthen psychological resilience. The core of prevention lies in building what might be termed “psychological immunity”—ensuring that the individual has a larger reservoir of buffers to draw upon when faced with stress.

I. Prevention Strategies: A Three-Tier Protective Net

1. Primary Prevention: Universal Measures

Establish regular daily rhythms

Maintain stable sleep (7–9 hours per night), with particular emphasis on a fixed wake-up time.

Eat a balanced diet, attending to nutrients such as omega-3 fatty acids, vitamin D, and B vitamins.

Obtain adequate daily sunlight, especially morning light, which helps regulate the circadian clock.

Regular physical activity

Aim for 150 minutes of moderate-intensity exercise per week (e.g., brisk walking, swimming).

Exercise stimulates the release of endorphins and dopamine, serving as a natural mood regulator.

Group exercise provides the dual benefits of physical activity and social support.

Making stress management routine

Learn to identify one’s personal stress signals (e.g., muscle tension, irritability, changes in sleep).

Build daily relaxation habits: deep breathing, mindfulness meditation, progressive muscle relaxation.

Set realistic expectations and avoid a chronic pattern of perfectionistic self-demands.

2. Secondary Prevention: Early Intervention for High-Risk Populations

Recognize warning signs: persistently low mood lasting more than two weeks, loss of interest, social withdrawal.

Following stressful life events (such as job loss, bereavement, or relationship breakdown), proactively increase self-care.

Individuals with a family history of depression should consider regular psychological “check-ups.”

Address subclinical depressive states promptly to avert progression to a full clinical episode.

3. Tertiary Prevention: Relapse Prevention During Recovery

Complete the full course of treatment; avoid discontinuing treatment prematurely once symptoms have abated.

Identify one’s personal early-warning signs of recurrence (e.g., specific thought patterns, behavioral shifts).

Develop an individualized relapse prevention plan in collaboration with a professional.

II. Self-Care: Actionable Daily Practices

1. Caring for Thoughts and Emotions

Cognitive balancing exercises: Record automatic negative thoughts and search for alternative explanations.

Making space for emotions: Acknowledge the validity of all emotional states and observe them without judgement.

Accumulating positive experiences: Each day, note three things that went well to cultivate an attitude of gratitude.

2. Maintaining Social Connection

Sustain at least one or two deep, supportive relationships.

Participate in community activities and interest-based groups to foster a sense of belonging.

Practice healthy communication: express needs, set boundaries, and ask for help.

3. Structuring Daily Life and Cultivating Meaning

Establish small daily rituals (e.g., a morning routine, a bedtime wind-down).

Set realistic, short-term goals to build a cumulative sense of mastery.

Engage in meaningful activities that connect to purposes larger than oneself.

4. Digital Habits for Mental Health

Set limits on screen time, particularly for social media.

Reduce blue-light exposure in the hour before sleep.

Curate one’s intake to include high-quality psychoeducational resources, and avoid self-diagnosis and misleading information.

III. Clarifying Important Concepts

Prevention is not about perfect control: Stress and low points are an unavoidable part of life. The goal of prevention is to strengthen resilience, not to eliminate all discomfort.

Self-care is not self-indulgence: It is a systematic, evidence-based practice, not a temporary escape from emotion.

Seeking help is a sign of strength: When self-regulation proves insufficient, reaching out promptly is a wise choice.

Mental health is a dynamic process: Like physical health, it requires ongoing maintenance rather than a one-off fix.

IV. When to Seek Professional Support

Professional assessment is recommended in the following circumstances:

Emotional distress persists for more than two weeks and interferes with daily functioning.

Thoughts of suicide or impulses to self-harm emerge.

Self-help efforts are ineffective and the situation continues to worsen.

Work, academic performance, or relationships are significantly compromised by emotional difficulties.

The heart of prevention and self-care is not the complete avoidance of low points, but ensuring that when storms come, you have already put up a sturdy umbrella. Mental health is like a garden; it needs daily watering and timely tending. Every small act of self-care begun today is a positive investment in your future mental health.

Original Chinese:

抑郁症的预防与自我关怀：守护心理健康的日常实践

理解预防：多层次、可实践的防护体系

抑郁症的发生涉及生物、心理、社会多重因素，虽然无法保证绝对不患病，但科学的生活方式与认知习惯可显著降低风险、增强心理韧性。预防的核心在于建立“心理免疫力”，使个体在面临压力时拥有更多缓冲资源。

一、预防策略：三级防护网

1. 一级预防：面向所有人

建立规律生活节律

保持稳定睡眠（7-9小时/天），特别是固定起床时间

均衡饮食，关注Omega-3、维生素D、B族维生素等营养素

每日适量日照，尤其早晨光照有助于调节生物钟

规律身体活动

每周150分钟中等强度运动（如快走、游泳）

运动产生内啡肽和多巴胺，是天然的情绪调节剂

团体运动兼具社交支持与身体活动双重益处

压力管理常态化

学习识别个人压力信号（如肌肉紧张、烦躁、睡眠变化）

建立日常放松习惯：深呼吸、正念冥想、渐进式肌肉放松

设置合理期待，避免长期“完美主义”自我要求

2. 二级预防：高风险人群的早期干预

识别风险信号：持续情绪低落超过两周、兴趣减退、社会退缩

在压力事件后（如失业、丧失、关系破裂）主动增加自我关怀

有抑郁症家族史者定期进行心理“体检”

及时处理“亚临床抑郁状态”，避免发展为临床抑郁

3. 三级预防：康复期防复发

完成完整的治疗疗程，避免症状缓解后过早停止治疗

识别个人复发预警信号（如特定思维模式、行为变化）

制定个性化复发预防计划，与专业人员协作

二、自我关怀：可操作的日常实践

1. 思维与情绪的自我照料

平衡思维练习：记录自动负面思维，寻找替代解释

情绪允许空间：承认所有情绪的合理性，不评判地观察它们

积极体验积累：每天记录3件好事，培养感恩心态

2. 社会连接维护

保持至少1-2个深度社会支持关系

参与社区活动、兴趣团体，建立归属感

练习健康沟通：表达需求、设立界限、寻求帮助

3. 生活结构与意义感

建立日常小仪式（如晨间惯例、睡前放松）

设定切实可行的短期目标，积累掌控感

参与有意义的活动，连接大于自我的目标

4. 数字心理健康习惯

设定屏幕时间限制，特别是社交媒体

睡前1小时减少蓝光暴露

关注优质心理教育资源，避免自我诊断和误导信息

三、重要观念澄清

预防不是完美控制：生活难免压力与低谷，预防目标在于增强韧性而非消除所有不适

自我关怀不是自我放纵：是基于科学证据的系统实践，而非暂时的情绪逃避

寻求帮助是力量的体现：当自我调节不足时，及时求助是智慧选择

心理健康是动态过程：如同身体健康，需要持续维护而非一劳永逸

四、何时需要专业支持

当出现以下情况，建议寻求专业评估：

情绪困扰持续超过两周且影响日常生活

出现自杀念头或自伤冲动

自我调节努力无效，状况持续恶化

工作、学习、人际关系因情绪问题严重受损

预防与自我关怀的核心不是完全避免低谷，而是在风雨来临时，你已为自己撑起一把结实的伞。 心理健康如同花园，需要日常浇灌与适时照料。今天开始的一小步自我关怀，都是对未来心理健康的积极投资。

**⑤ Topic on social support and recovery:**

Social Support and Communication in Depression: How to Be an Effective Supporter

Understanding the Importance of Social Support

A robust body of scientific evidence identifies strong social support as one of the most critical protective factors in the course of recovery from depression. Effective support not only alleviates the sense of isolation but also measurably improves treatment adherence and overall recovery outcomes. Conversely, support that is well-intentioned but misapplied can inadvertently cause harm.

I. Principles of Effective Communication for Supporters

1. Listening Takes Precedence Over Advising

Deep listening: Give the person full space to express what they are experiencing without rushing to interrupt or offer solutions.

Validating the feeling: “That sounds truly difficult,” rather than “You should try to look on the bright side.”

Suspending judgement: Refrain from responding to emotional distress within a framework of right or wrong.

2. Choosing Words Carefully: Supportive vs. Harmful Expressions

Helpful things to say:

“I’m here with you.”

“This must be so hard for you.”

“Would you like to talk about it? It’s completely fine if you don’t.”

“Let’s work through this together.”

Statements to avoid:

“You’re just overthinking everything.”

“Other people have it much worse than you.”

“You just need to pull yourself together.”

“What do you even have to be depressed about?”

3. Acceptance and Patience

Accept that the person’s presentation—profound fatigue, irritability, social withdrawal—are manifestations of the illness itself.

Understand that recovery is not linear; allow for days that feel like steps backward, and do not exert “progress pressure.”

Maintain steady, consistent support even when no obvious improvement is visible in the short term.

II. A Practical Guide to Supportive Actions

1. Daily Companionship

Low-demand companionship: Engage in quiet, shared activities (taking a walk, listening to music) without insisting on conversation.

Assistance with daily tasks: Help with grocery shopping, cooking, or other chores to lessen the burden of everyday responsibilities.

Accompanying to appointments: Help schedule visits, write down medical instructions, and attend follow-up consultations.

2. Treatment Support

Educate yourself about the fundamentals of depression and what the treatment process entails.

Assist in noticing medication side effects, but take care not to assume the role of an “overseer.”

Encourage treatment while respecting the individual’s choices; do not press unproven remedies or folk cures.

3. Crisis Recognition and Response

Recognizing warning signs:

Talking about death or making a specific suicide plan.

A sudden, unexplained brightening of mood (which may indicate a decision to end the suffering).

Putting affairs in order, giving away treasured possessions.

How to respond:

Ask directly: “Are you having thoughts of harming yourself?”

Take every mention of suicide seriously.

Immediately connect with professional resources (a mental health crisis line, an emergency department).

Do not promise confidentiality where safety is at stake; safety takes precedence.

III. Building a Family Support System

1. Adjusting Family Communication

Hold regular family conversations, framing the situation as “we vs. the depression” rather than “we vs. you.”

Express care and boundaries clearly: “I care about you, and I also need to look after my own well-being.”

2. Division of Responsibilities

Avoid placing the entire burden of support on a single family member; divide tasks in a reasonable and sustainable way.

Consider bringing in outside support, such as rotating among relatives and friends, or accessing professional respite services.

3. Self-Care for the Supporter

Supporters require support themselves: joining a family support group can be invaluable.

Set healthy boundaries: be clear about what you are able to offer and where your limits lie.

Attend to your own emotional state, and actively maintain your own social support network.

IV. Support Within the Workplace and Community

1. Workplace Support

Offer flexible work arrangements where feasible, such as a temporary adjustment of duties.

Cultivate a mental-health-friendly culture that reduces stigma.

Ensure access to professional resources such as Employee Assistance Programs.

2. Community Support Networks

Help the person connect with peer support groups for depression.

Assist in locating reliable, evidence-based mental health resources.

Create an inclusive social environment that allows for “phased participation” rather than demanding full engagement.

V. Important Reminders: Boundaries and the Ethics of Supporting

You are not the therapist: Offer support, but do not attempt to substitute for professional treatment.

Respect autonomy: Even when you hold a different view, respect the person’s decisions about their own care.

Do not accept abuse: Depression is never a legitimate excuse for verbal or emotional abuse.

Protect yourself: When the demands of the supporter role begin to compromise your own health, seek professional guidance for yourself.

VI. When You Are the Person with Depression: How to Seek Support Effectively

Make requests concrete: Say “I need someone just to sit with me for a little while” rather than a general “I need help.”

Share educational material: Provide reliable information about depression to help others understand what you are experiencing.

Allow others to be imperfect: Give your supporters the space to learn and, occasionally, to get things wrong.

Diversify your sources of support: Avoid relying entirely on a single individual.

True support is not about pulling someone out of the abyss; it is about climbing down to sit beside them, so they know they do not have to face the darkness alone. The quality of social support, more often than not, resides not in eloquent words but in a steady, non-judgmental presence. Whether you are the supporter or the person receiving support, building a healthy, balanced support relationship is itself an essential component of the recovery process.

Note: If you or someone you care about is currently in crisis, please contact a local mental health crisis line immediately or go to the nearest hospital emergency department. Professional help is always available; you do not have to carry this alone.

Original Chinese:

抑郁症的社会支持与沟通：如何成为有效的支持者

理解社会支持的重要性

科学证据表明，良好的社会支持是抑郁症康复过程中最关键的保护因素之一。有效的支持不仅能减轻患者的孤独感，还能显著提高治疗依从性和康复效果。然而，不当的支持方式可能无意中造成伤害。

一、支持者的有效沟通原则

1. 倾听优于建议

深度倾听：给予完整表达空间，不急于打断或提供解决方案

确认感受：“听起来这真的很艰难”，而非“你应该往好处想”

避免评判：不用“对错”框架回应情绪体验

2. 用对语言：支持性 vs. 伤害性表达

有效表达：

“我在这里陪着你”

“这对你一定很不容易”

“你想聊聊吗？不想说也没关系”

“我们一起想办法”

避免表达：

“你就是想太多了”

“别人比你更惨”

“振作起来就好了”

“这有什么好抑郁的”

3. 接纳与耐心

接受患者症状表现：无力、易怒、社交回避都是症状的一部分

理解康复非线性：允许有“倒退”的日子，不施加“进步压力”

保持稳定支持：即使短期内看不到明显改善

二、具体支持行动指南

1. 日常陪伴

低压力陪伴：一起进行安静活动（散步、听音乐），不强制交谈

协助日常事务：帮忙采购、烹饪，减轻基本生活压力

陪伴就医：帮助预约、记录医嘱、陪同复诊

2. 治疗支持

了解抑郁症基本知识，理解治疗过程

协助监测药物副作用，但不扮演“监督者”

鼓励但尊重治疗选择，不强行推荐偏方

3. 危机识别与应对

识别风险信号：

谈论死亡或自杀的具体计划

突然的情绪“好转”（可能是决定结束痛苦的信号）

安排后事、赠送珍贵物品

如何回应：

直接询问：“你是否有伤害自己的想法？”

认真对待所有自杀言论

立即联系专业资源（心理危机热线、急诊）

不承诺保密，安全优先

三、家庭支持系统建设

1. 家庭沟通调整

定期家庭会议，以“我们vs.抑郁症”而非“我们vs.你”的立场

明确表达关心与界限：“我关心你，也需要照顾好自己”

2. 责任分工

避免一人承担所有支持责任，家庭内部合理分工

考虑引入外部支持（亲友轮换、专业喘息服务）

3. 自我关怀

支持者也需要支持：加入家属支持团体

设置健康界限：明确自己能做和不能做的

关注自身情绪：寻求自己的社会支持

四、工作与社区环境支持

1. 工作场所支持

提供灵活的工作安排（如暂时调整职责）

建立心理健康友好文化，减少病耻感

提供员工援助计划等专业资源

2. 社区支持网络

帮助连接抑郁症支持团体

协助寻找可靠的心理健康资源

创建包容的社交环境，允许“阶段性参与”

五、重要提醒：支持的界限与伦理

你不是治疗师：提供支持，但不替代专业治疗

尊重自主权：即使不同意，也尊重患者的治疗决定

不接受虐待：抑郁症不是言语或情感虐待的借口

保护自己：当支持危及自身健康时，寻求专业指导

六、当你是抑郁症患者：如何有效寻求支持

具体表达需求：“我需要有人陪我坐一会儿”而非“我需要帮助”

准备信息资料：分享可靠的抑郁症资料，帮助他人理解

允许他人不完美：给予支持者学习空间

多样化支持源：不依赖单一个人

真正的支持不是把某人拉出深渊，而是下去陪伴，让他们知道不必独自面对黑暗。 社会支持的质量往往不在于华丽的言辞，而在于持续存在的、不带评判的陪伴。无论作为支持者还是被支持者，建立健康、平衡的支持关系本身就是康复过程的重要组成部分。 注：如果您或您关心的人正处于危机中，请立即联系当地心理危机干预热线或前往最近医院急诊科。专业帮助随时可得，您不必独自承受。
